# Rare-Earth–Silyl ate-Complexes Opening a Door to Selective Manipulations

**DOI:** 10.1021/acs.inorgchem.1c00904

**Published:** 2021-05-25

**Authors:** Alexander Pöcheim, Christoph Marschner, Judith Baumgartner

**Affiliations:** Institut für Anorganische Chemie, Technische Universität Graz, Stremayrgasse 9, 8010 Graz, Austria

## Abstract

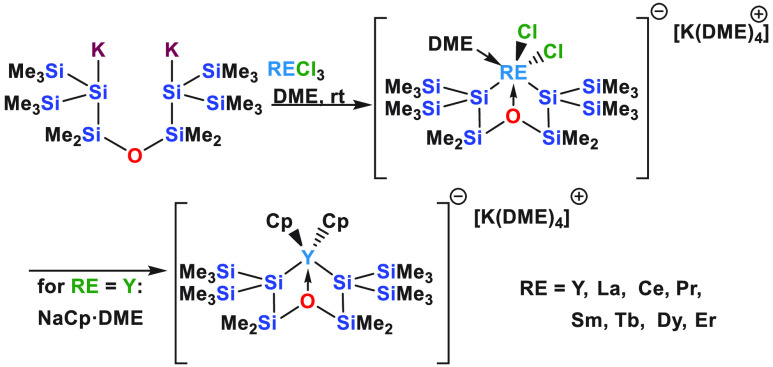

The reactions of a number of rare-earth (RE) trichlorides and an oligosilanylene diide containing a siloxane unit in the backbone in DME are described. The formed products of the type [(DME)_4_·K][(DME)·RE(Cl)_2_{Si(SiMe_3_)_2_SiMe_2_}_2_O] (RE = Y, La, Ce, Pr, Sm, Tb, Dy, and Er) are disilylated dichloro metalate complexes and include the first examples of Si–La and Si–Pr compounds as well as the first structurally characterized example of a Si–Dy complex. A most intriguing aspect of the synthesis of these complexes is that they offer entry into a systematic study of the still largely unexplored field of silyl RE complexes by the possibility of ligand exchange reactions under preservation of the Si–RE interaction. This was demonstrated by the conversion of [(DME)_4_·K][(DME)·RE(Cl)_2_{Si(SiMe_3_)_2_SiMe_2_}_2_O] to [(DME)_4_·K][Cp_2_Y{Si(SiMe_3_)_2_SiMe_2_}_2_O].

## Abstract 2

Reactions of some of rare-earth (RE) trichlorides with a siloxane containing oligosilanylene diide gave products of the type K[Cl_2_RE{Si(SiMe_3_)_2_SiMe_2_}_2_O(DME)] (RE = Y, La, Ce, Pr, Sm, Tb, Dy, and Er) including the first examples of Si−La and Si−Pr compounds. These complexes provide opportunities for a systematic study of silyl RE complexes offering the possibility of ligand exchange reactions under preservation of the Si−RE interaction, as was demonstrated by the conversion of K[Cl_2_Y{Si(SiMe_3_)_2_SiMe_2_}_2_O(DME)] to K[Cp_2_Y{Si(SiMe_3_)_2_SiMe_2_}_2_O].

## Introduction

The chemistry of transition metal silyl complexes started in the 1950s,^1^ but it required some 30 years until this field reached the status of an established research area.^2^ Nowadays, numerous silyl complexes of early and late transition metals are known, and we have a fairly good understanding of the bonding interactions between silicon and transition metals. Examples comprise single, double, and even triple Si–metal bonds.^2,3^

The chemistry of rare-earth (RE; group 3 and lanthanides, Ln) metal silyl complexes^4^ was first investigated by Schumann and co-workers in the mid-1980s,^5−7^ followed by significant contributions by the Tilley group in the 1990s.^8−12^ Some years ago our group has entered the field and we have contributed a few examples of lanthanide complexes with mono- or bidentate oligosilanyl ligands.^13−17^

A recent review by Réant, Liddle, and Mills, which does an excellent job describing the development of the field, listed 54 structurally characterized examples with RE–silicon bonds,^18^ suggesting that RE silyl chemistry has reached a degree of maturity. The number of 54 examples is still not very high, given that there are 17 RE elements. A more detailed analysis reveals that 30 examples of the 54 include the two elements samarium and ytterbium and that there are no reported examples of silyl complexes for the elements lanthanum (La), praseodymium (Pr), and promethium (Pm), and that there is no reported structural proof for silyl complexes of neodymium (Nd), dysprosium (Dy), and erbium (Er). The strong predominance of Sm and Yb can be explained by the fact that most of these complexes feature the metals in the oxidation state +2, which for some reason seemed to be the greater focus of researchers. However, the ease of formation of silyl complexes of the remaining 14 elements (if the radioactive promethium is excluded) seems not to be equal for all elements. Especially, different propensities for crystallization can be observed. While crystallinity is not necessarily a prerequisite for the characterization of chemical compounds, it is definitely an advantage for paramagnetic compounds with coordinating solvent molecules, which can pose a challenge to routine NMR characterization.

One aspect of our recent studies on RE silyl complexes was concerned with the search for silyl ligands containing additional donor sites to satisfy the coordination requirements of RE elements.^15,16^ In particular, the use of a 3-oxa-1,5-oligosilanylene dianion was found useful for the preparation of Yb(II) and Sm(II) complexes with THF molecules occupying three positions of octahedrally coordinated lanthanide ions. For the current study, we were interested to explore the potential of the 3-oxa-1,5-oligosilanylene ligand for Ln(III) ions.^15^

Frequently, reactions of silanides with Ln(III) compounds lead to the formation of ate-complexes. This was already observed by Schumann and co-workers in their reactions of Na[Cp_2_LnCl_2_] (Ln = Dy, Ho, Er, Tm, and Lu) with 2 equiv of Me_3_SiLi to Li[Cp_2_Ln(SiMe_3_)_2_]^5−7^ and later confirmed by us in reactions of Cp_3_Ln with (Me_3_Si)_3_SiK, which led to K[Cp_3_LnSi(SiMe_3_)_3_] ate-complexes for the elements Ce, Sm, Gd, Ho, and Tm.^17^ Reacting Cp_3_Ln with K(Me_3_Si)_2_Si(SiMe_2_)_2_Si(SiMe_3_)_2_K^19,20^ led to the cyclic disilylated products K[Cp_2_Ln{Si(SiMe_3_)_2_SiMe_2_}_2_] via elimination of CpK for Ln = Sm, Gd, Tb, Ho, and Tm.^13,17^ The reaction of the oligosilanylene diide with Cp_3_Ce took a different course to the acyclic complex K_2_[Cp_3_Ce(Me_3_Si)_2_Si(SiMe_2_)_2_Si(SiMe_3_)_2_CeCp_3_].^13^

Of all reported RE silyl complexes, only five examples are known to contain halide ligands. These five examples are restricted to the elements Y and Gd. Sgro and Piers reported reactions of YI_3_·(THF)_3.5_ and GdI_3_·(THF)_3.5_ with R(Me_3_Si)_2_SiK (R = SiMe_3_ and Et) to R(Me_3_Si)_2_SiYI_2_·(THF)_3_ and R(Me_3_Si)_2_SiGdI_2_·(THF)_3_.^21^ The remaining example was reported by Sadow and co-workers, who showed that (Me_2_HSi)_3_SiK and YCl_3_ give K_2_(Et_2_O)_2_[Y{Si(SiHMe_2_)_3_}_2_Cl_3_(Et_2_O)], which rapidly decomposes at room temperature in solution or in the solid state to several unidentified silyl-containing species.^22^

In the present study, we report the preparation of a number of RE complexes with a bidentate oligosilanylene ligand, including examples with Y, La, Ce, Pr, Sm, Tb, Dy, and Er. These comprise the first examples of Si–La and Si–Pr compounds and the first structurally characterized example of a Si–Dy complex.

## Results and Discussion

### Synthesis of Dichlorodisilyl RE ate-Complexes

**Scheme 1:**
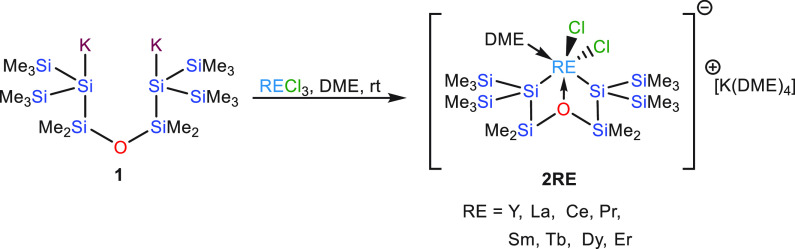
Synthesis of Disilylated Rare-Earth Dichloro ate-Complexes **2Y**, **2La**, **2Ce**, **2Pr**, **2Sm**, **2Tb**, **2Dy**, and **2Er**

While previous attempts to react RE chlorides (RECl_3_) with the oligosilanylene diide K(Me_3_Si)_2_Si(SiMe_2_)_2_Si(SiMe_3_)_2_K^19,20^ were not successful, we decided to switch to the mentioned 3-oxa-1,5-oligosilanylene ligand with K(Me_3_Si)_2_SiSiMe_2_OSiMe_2_Si(SiMe_3_)_2_K (**1**) as the reagent, where the siloxane oxygen offers an additional coordination possibility for the RE element. We thus carried out reactions of RECl_3_ (RE = Y, La, Ce, Pr, Sm, Tb, Dy, and Er) with **1** in DME (Scheme 1). Starting the series with YCl_3_, we obtained the separated ion pair [(DME)_4_·K][(DME)·Y(Cl)_2_{Si(SiMe_3_)_2_SiMe_2_}_2_O] (**2Y**) with the potassium ion coordinated by four DME molecules.

Only a small number of yttrium silyl complexes are known.^9,21−23^ Two examples of these were found to decompose at ambient temperature and no NMR data are available. This is unfortunate since ^89^Y is a spin 1/2, 100% nucleus and Y(III) complexes are diamagnetic and therefore NMR spectroscopically accessible. The ^29^Si NMR spectrum of **2Y** features a signal at −161.6 ppm for the ligating silicon atoms and a ^1^*J*_Si–Y_ coupling constant of 38 Hz. The chemical shift of the resonance clearly indicates a very high degree of polarity of the Si–Y interaction, which is only slightly less polar than the respective magnesium compound [(DME)·Mg{Si(SiMe_3_)_2_SiMe_2_}_2_O] (δ_^29^Si_ = −166.7 ppm).^16^ Such strong silanide character was neither observed for Tilley’s Cp*_2_YSiH(SiMe_3_)_2_ (δ_^29^Si_ = −120.0 ppm, ^1^*J*_Si–Y_ = 92 Hz)^9^ nor for Piers’ R(Me_3_Si)_2_SiYI_2_·(THF)_3_ (R = SiMe_3_: δ_^29^Si_ = −134.7 ppm, ^1^*J*_Si–Y_ = 63 Hz; R = Et: δ_^29^Si_ = −73.5 ppm, ^1^*J*_Si–Y_ = 71 Hz),^21^ both of which are neutral complexes. The expected higher degree of covalent Si–Y interaction for the neutral compounds is nicely reflected by their larger ^*1*^*J*_Si–Y_ coupling constants.

**Figure 1:**
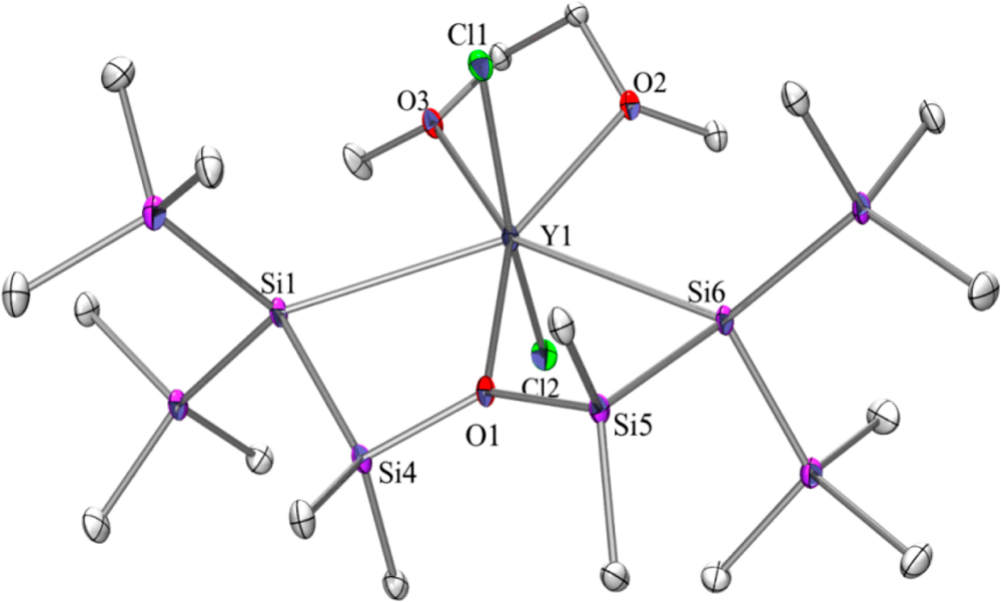
Molecular structure of **2Y** (thermal ellipsoid plot drawn at the 30% probability level). Only the anionic part is shown. Structures of **2Ce**, **2Pr**, **2Sm**, **2Tb**, and **2Dy** are isotypic to **2Y**. ORTEP plots of all isotypic structures together with selected bond distances and angles can be found in the Supporting Information.

The solid state structure of **2Y** (Figures 1 and S1, Table 1), which crystallizes in the triclinic space group *P*1̅ was determined by single-crystal XRD analysis. The coordination geometry around Y in the negatively charged part of the complex is a distorted pentagonal bipyramid with the chlorides as apexes and the disilanide ligand and the DME molecule in the equatorial plane. The Si–Y distances of 3.0575(13) and 3.0641(13) Å (Table 1) are longer than those reported so far. Piers’ neutral compounds (Me_3_Si)_3_SiYI_2_·(THF)_3_ (*d*_Si–Y_ = 2.979(3) Å) and (Me_3_Si)_2_EtSiYI_2_·(THF)_3_ (*d*_Si–Y_ = 2.9611(18) Å)^21^ feature shortened distances reflecting the more covalent interaction. While Evans’ K[Cp’_3_YSiH_2_Ph]^23^ is also an ate-complex, the steric demand of the SiH_2_Ph ligand is markedly smaller, favoring a shorter Si–Y distance of 2.953(1) Å. To a smaller extent, this argument is also true for the SiH(SiMe_3_)_2_ ligand used by Sadow and co-workers; nevertheless, the Si–Y distance determined for K_2_(Et_2_O)_2_[Y{Si(SiHMe_2_)_3_}_2_Cl_3_(Et_2_O)]^22^ of 3.030(1) Å is close to that found for **2Y**. It is interesting to note that the respective distance in Lappert’s Cp_3_Y–NHSi (*N*-heterocyclic silylene) complex^24^ is very similar (3.038(2) Å).

**Table 1:** Selected Bond Distances (Å) and Bond Angles (deg) for **2Y**, **2Ce**, **2Pr**, **2Sm**, **2Tb**, **2Dy**, and **5**

	Si–RE	O_Si_–RE	O_DME_–RE	Cl–RE	Si–RE–Si
**2Y**	3.064(2)	2.422(3)	2.443(3)	2.584(1)	133.07(4)
	3.057(1)		2.534(3)	2.585(1)	
**2Ce**	3.144(2)/	2.575(3)	2.632(5)	2.688(2)	129.49(4)
	3.159(2)		2.613(4)	2.709(2)	
**2Pr**	3.119(2)/	2.586(6)/	2.575(7)	2.678(3)	130.27(7)/
	3.125(2)/	2.508(5)	2.655(7)	2.683(3)	130.49(6)
	3.167(2)/		2.615(6)	2.681(3)	
	3.159(2)		2.632(7)	2.684(3)	
**2Sm**	3.089(1)/	2.501(2)	2.528(2)	2.649(1)	131.63(2)
	3.099(1)		2.596(3)	2.6390(9)	
**2Tb**	3.073(2)/	2.469(3)	2.565(5)	2.610(2)	132.70(4)
	3.081(2)		2.501(5)	2.621(2)	
**2Dy**	3.077(3)/	2.45(1)	2.55(1)	2.596(3)	132.96(9)
	3.077(4)		2.516(9)	2.603(3)	
**5** (Y)	3.1315(9)/	2.484(2)	n.a.	n.a.	131.03(2)
	3.1459(9)				

A comparison of complex **2Y** with [YCl_3_·(DME)_2_]^25^ shows that the Y–Cl distances of **2Y** (2.584(1)/2.585(1) Å) are slightly shorter, whereas the Y–O_DME_ distances (2.443(3)/2.534(3) Å) are somewhat longer than in [YCl_3_·(DME)_2_] (*d*_Y–Cl_: 2.597(2)–2.600(2) Å, *d*_Y–O_: 2.379(3)–2.457(3) Å). It is also interesting to note that there is almost no difference between the Y–O_DME_ distances and that to the less basic siloxane oxygen atom: Y–O_(SiMe2)2_ (2.422(3) Å) in **2Y**.

The complex cation [K(DME)_4_]^+^ present in **2Y** is a frequently observed counterion in main group,^26−29^ transition metal,^30−33^ and f element^34,35^ chemistry. Its popularity is maybe associated with the facts that the coordination sphere of potassium is very well covered; thus, the formed complex ion is almost spherical which frequently facilitates crystallization. Anions with negatively charged B,^26^ Si,^27,28^ or Ga^29^ atoms as well as ate-complexes of Cr,^30^ Fe,^31,32^ Co,^33^ Yb,^34^ and U^35^ were structurally characterized with [K(DME)_4_]^+^ as counterion.

While there are the mentioned few examples of yttrium silyl compounds, we are not aware of any lanthanum silyl complexes. Apart from some theoretical treatment in the context of Si–H activation chemistry,^36,37^ we found no further mentioning of silyl lanthanum compounds. This is surprising as La shares with Y the property that its trivalent complexes are diamagnetic and thus susceptible to NMR spectroscopy.

The reaction of oligosilanylene diide **1** with LaCl_3_ proceeded cleanly as judged by NMR spectroscopic analysis (Scheme 1). Unfortunately, the ^29^Si NMR spectrum showed only signals for the SiMe_2_ (36.9 ppm) and SiMe_3_ (−5.7 ppm) groups, and no signal for the metalated silicon atom could be detected. However, the shifts of the two observed signals are very similar to what was found for **2Y**, indicating a strong silanide character and an interaction of the siloxane oxygen with the lanthanum atom. The same is true for the ^1^H and ^13^C signals of **2La**, which correspond to the analogous signals of **2Y**.

Despite the widespread assumption that the chemistry of RE metals is very similar for all of these elements, we have frequently found that the ease of reaction can be quite different for different metals and that the physical properties of complexes such as solubility can cover a wide range. For these reasons we were not too surprised that we were unable to obtain suitable crystals for structure analysis of **2La**. While this is unfortunate, we hope that derivatization of **2La**, as shown below for **2Y**, will eventually help us to obtain a first example of a structurally characterized silyl lanthanum complex.

Only two examples of silyl cerium compounds have been reported so far.^13,17^ Both complexes are silyl ate-complexes of Cp_3_Ce. The respective Si–Ce distances of K[Cp_3_CeSi(SiMe_3_)_3_] and K_2_[{Cp_3_CeSi(SiMe_3_)_2_SiMe_2_}_2_] are 3.1551(19) and 3.2283(2) Å, respectively. Reaction of **1** with CeCl_3_ proceeded in a way that crystals of **2Ce** could be obtained (Scheme 1).

As found for **2Y**, also complex **2Ce** crystallizes in the triclinic space group *P*1̅ with very similar cell parameters (Table S1). However, while we could measure compound **2Y** at a temperature of 100 K, we had to raise the temperature for **2Ce** to 200 K to obtain adequate diffraction data. Its structure in the crystal (Figure S2, Table 1) is very similar to that of **2Y**, but of course all bonds to Ce are accordingly elongated. The Si–Ce distances of 3.1442(16) and 3.1593(16) Å (Table 1) are close to what we observed for K[Cp_3_CeSi(SiMe_3_)_3_].^17^ Comparison with a DME complex of CeCl_3_ is also possible, but due to the larger ionic radius of Ce, this complex exists in a dimeric state with two bridging chloride ligands as [CeCl_3_·(DME)_2_]_2_.^38^ While the Ce–Cl (2.688(2)/2.709(2) Å) distances of **2Ce** are somewhat shorter, the respective Ce–O (2.632(5)/2.613(4) Å) distances are quite similar to that of [CeCl_3_·(DME)_2_]_2_ (*d*_Ce–Cl_: 2.728(1)/2.770(1) Å (nonbridging Cl), *d*_Ce–O_: 2.584(3)–2.666(4) Å).

Attempts to obtain NMR data of **2Ce** were met by a surprise. While for our previously reported silyl cerium compounds^13,17^ not even ^1^H NMR spectra could be obtained, the respective spectra of **2Ce** look surprisingly decent. The slightly broadened ^1^H NMR signals for the singlets of the SiMe_3_ and SiMe_2_ groups were detected at −0.32 and 1.16 ppm, respectively. We do not exactly know how to explain this different behavior, but two possible explanations seem plausible. The first one assumes that the configurational lock of the cyclic structure of **2Ce** versus the rotational freedom of the silyl ligands in our previous examples, plays a decisive role. A structurally similar Ce(III) compound reported by Liddle and co-workers showed that the chemical shifts of ligand parts above or below the equatorial plane were not much affected by the paramagnetic influence.^39^ Another possible explanation is that the situation in solution might feature some degree of dissociation between the RE and silanide fragments, so that the paramagnetic influence of the Ce fragment of the silyl ligand could be diminished.

There are no reported examples for praseodymium silyl complexes so far. The substance class was however assessed theoretically in a DFT study by Perrin, Maron, and Eisenstein, where they investigated the SiH_4_ activation chemistry of Cp_2_LnH.^36^ To obtain the first example of a praseodymium silyl compound, we treated oligosilanylene diide **1** with PrCl_3_ in DME (Scheme 1). The reaction allowed us to isolate **2Pr** as green crystals. Two very broad signals could be observed in the ^1^H NMR spectrum, which we tentatively assign to the CH_2_/CH_3_ resonances of DME and the CH_3_ signals of the SiMe_3_ and SiMe_2_ groups. The crystals of **2Pr**, which crystallize in the monoclinic space group *P*2_1_/*c*, could be subjected to XRD analysis (Figure S3, Table 1). The quality of the structure solution is not very good, but two independent molecules in the asymmetric unit cell could be found. The two molecules feature different Si–Pr distances of 3.119(2)/3.125(2) and 3.167(2)/3.159(2) Å. The average value of 3.1425 Å is slightly shorter than the values observed for **2Ce** (3.1515 Å), consistent with the reported difference of the covalent radii of Ce and Pr (2.04 and 2.03 Å).^40^ The DME complex of PrCl_3_ is similar to that of CeCl_3_ and exists as the Cl-bridged dimer [PrCl_3_·(DME)_2_]_2_ in the solid state.^38^ Again, the Pr–Cl distances of **2Pr** (2.678(3)–2.684(3) Å) are noticeably shorter than the respective interactions between Pr and the nonbridging Cl ligands of [PrCl_3_·(DME)_2_]_2_, whereas the Pr–O_DME_ distances of **2Pr**, covering a range between 2.575 and 2.655(7) Å, are in accordance with the 2.587 Å distance found in [PrCl_3_·(DME)_2_]_2_.

With the good experiences concerning formation of samarium silyl complexes, the reaction of oligosilanylene diide **1** with SmCl_3_ to give **2Sm** as deep purple crystals is probably the least unexpected one in this series. Surprisingly, the compound gave a decent ^29^Si NMR spectrum with barely shifted resonances at +42.9 and −9.0 ppm and no signal for the metalated silicon atoms. Again, the reasons for this behavior are not obvious.

Similar to **2Y** and **2Ce**, also complex **2Sm** crystallizes in the triclinic space group *P*1̅ (Table S1). The structural features of **2Sm** (Figure S4, Table 1) follow the same pattern as observed for the complexes discussed above. The Sm–Si distances of 3.0891(11) and 3.0987(10) Å clearly show the oxidation state of Sm as Si–Sm(II) bonds are typically found between 3.15 and 3.23 Å.^18^ Of all reported Si–Sm(III) complexes, only [{K(18-crown-6)}_2_Cp][Cp_3_Sm{Si(SiMe_3_)_3_}] (3.1013(17) Å)^17^ exhibited a bond longer than those in **2Sm**. The DME adduct of SmCl_3_ [SmCl_3_·(DME)_2_] is monomeric,^41^ and its Si–Cl distances (2.647(1)–2.656(1) Å) are only barely longer than those of **2Sm** (2.649(1)/2.6390(9) Å). The Sm–O distances of **2Sm** are 2.528(2) and 2.596(3) Å, while the respective distances in [SmCl_3_·(DME)_2_] range from 2.448(3) to 2.534(3) Å.

The only structurally characterized example of a terbium silyl complex reported so far is the terbatetrasilacyclopentane ate-complex [{K(18-crown-6)}_2_Cp] [Cp_2_Tb{[Si(SiMe_3_)_2_SiMe_2_]_2_}],^13^ and a terbium silyl complex was also included in a theoretical study.^36^ The colorless crystals of compound **2Tb** resulting from the reaction of **1** with TbCl_3_ in DME (Scheme 1) crystallized again in the triclinic space group *P*1̅, and the picture of the structure (Figure S5, Table 1) is similar to what we found for the other compounds of the same type. The Si–Tb distances of 3.0733(18) and 3.0814(17) Å are significantly longer than the 3.0189(26) Å distance observed for the mentioned terbatetrasilacyclopentane.^13^ This is not entirely unexpected as a similar elongation is also present when comparing the respective samaratetrasilacyclopentane^17^ to **2Sm**. The comparison of **2Tb** with [TbCl_3_·(DME)_2_]^38^ reveals that the Tb–Cl distances of **2Tb** (2.610(2)/2.621(2) Å) are almost identical to that of [TbCl_3_·(DME)_2_] (2.610(1)–2.617(1) Å). The Tb–O distances of **2Tb** (2.565(5)/2.501(5) Å) are, however, somewhat elongated compared to those of [TbCl_3_·(DME)_2_] (2.405(4)–2.500(4) Å).

No structurally characterized example of a dysprosium silyl complex has been reported so far. The only example of this class of compounds that has been reported is [Li(DME)_3_][Cp_2_Dy(SiMe_3_)_2_], which was prepared by Schumann et al. more than 30 years ago.^6^ Interestingly enough, only recently Gao and co-workers reported the synthesis of dysprosium germyl and stannyl complexes [(C_5_H_4_*^i^*Pr)_2_Dy(GePh_3_)(THF)] and [Cp*_2_Dy(SnPh_3_)(THF)].^42^ The absence of a structurally characterized Si–Dy complex seems a bit odd. A likely explanation for this is that the silyl complexes of different RE metals frequently exhibit fairly different crystallization propensities. Given the difficulties in getting conclusive NMR characterization and the problems in obtaining elemental analysis data, unambiguous identification of these complexes is a challenge. It can probably be assumed that attempts for the synthesis of silyl dysprosium compounds have been undertaken but were not reported due to the mentioned problems. This assumption is certainly true for our group, and we are glad that the ligand derived from dianion **1** provided us with a crystalline sample of **2Dy** (Scheme 1). The yellow crystals of **2Dy** (Figure S6, Table 1) follow the other examples of the series (except for **2Pr**), crystallizing in the triclinic space group *P*1̅. Both Si–Dy bonds amount to 3.077(3) Å, much longer than the Ge–Dy distance in [(C_5_H_4_*^i^*Pr)_2_Dy(GePh_3_)(THF)] (2.981(1) Å),^42^ exemplifying once again how dependent on steric conditions these distances are. The comparison of the Dy–Cl and Dy–O_DME_ distances of **2Dy** (*d*_Dy–Cl_ = 2.596(3)/2.603(3) Å; *d*_Dy–O_ = 2.55(1)/2.516(9) Å) with [DyCl_3_(DME)_2_] (*d*_Dy–Cl_ = 2.5964(13)–2.5984(13) Å; *d*_Dy–O_ = 2.395(3)/2.472(3) Å)^43^ continues the trend from **2Tb**. The Dy–Cl bonds of both complexes are almost identical, while the Dy–O distances of **2Dy** are significantly longer.

The situation for erbium silyl complexes is similar to that of Dy–Si compounds. No structurally characterized example of an erbium silyl complex has been reported so far. The only known example is [Li(DME)_3_][Cp_2_Er(SiMe_3_)_2_],^6^ which was reported by Schumann et al. in the same study together with [Li(DME)_3_][Cp_2_Dy(SiMe_3_)_2_]. Our attempt to prepare **2Er** followed the same strategy as for all other compounds described above (Scheme 1). The reaction proceeded, and we were able to obtain pinkish crystals, which, however, were not suitable for XRD analysis. The substance was found to be very light-sensitive, and the main reason that we are confident to have prepared **2Er** is that it was possible to measure a ^1^H NMR spectrum featuring the typical paramagnetically shifted signals.

### Reactivity of **2Y** toward InCl_3_ and NaCp

Meanwhile, several silyl RE complexes have been prepared; the number of instances where basic reactivity patterns have been established is much smaller. Examples of reactions under preservation of the Si–RE bond are very scarce.

**Scheme 2:**
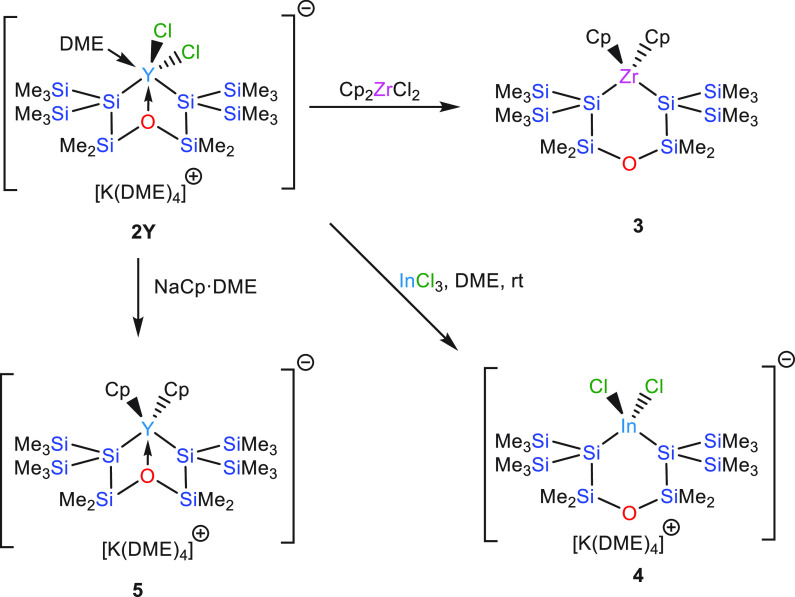
Reactions of the Disilylated Yttrium ate Complex **2Y** with Cp_2_ZrCl_2_, InCl_3_, or NaCp to Cyclic Disilylated Zirconocene **3**, Indium-Containing ate-Complex **4** or Disilylated Yttrocene ate-Complex **5**

As outlined above, the silanide character of the silyl ligands of these compounds in general and that of the oligosilanylene ligand in the **2RE** complexes in particular, is substantial. We have previously demonstrated an application of this property by reaction of a neutral ytterba(II)tetrasilacyclopentane with zirconocene dichloride to obtain a zirconatetrasilacyclopentane.^14^ In the course of the present study, we were interested in whether RE(III)–Si ate-complexes can also react as silyl transfer reagents. For this reason, we treated **2Y** with Cp_2_ZrCl_2_ and obtained 1-zircona-4-oxatetrasilacyclohexane **3** (Scheme 2), which had been prepared previously in the reaction of **1** with Cp_2_ZrCl_2_.^16^ Another reaction of **2Y** with InCl_3_ gave the respective cyclic indium compound, **4** (Scheme 2). Previously, it had been shown that the reactions of oligosilanylene diides with InCl_3_ and GaCl_3_ lead to formation of cyclic dichloroindate or -gallate complexes.^44^ The ^29^Si NMR spectrum of **4** features signals at 13.5, −6.4, and −131.8 ppm. Compared to **2Y** (δ_^29^Si_ = 37.7, −6.5, −161.6 ppm), **1** (δ_^29^Si_ = 27.6, −7.0, −185.7 ppm),^15^ and [(Me_3_Si)_3_SiSiMe_2_]_2_O (δ_^29^Si_ = 13.4, −10.5, −132.8),^15^ it is evident that the Si–In interaction of **4** is rather covalent as can be assumed from the downfield-shifted central silicon resonance at δ_^29^Si_ = −131.8 ppm. The analogous signal for an indate embedded into a five-membered cyclosilane was detected at δ_^29^Si_ = −113.4 ppm,^44^ which is similar to the resonances for the acyclic compound Li(THF)_2_[Cl_2_In{Si(SiMe_3_)_3_}_2_]^45^ at −6.4 and −113.1 ppm.^46^ The fact that the SiMe_2_O resonance of **4** is found at 13.5 ppm suggests no interaction of the oxygen with the indium. Unfortunately, **4** is difficult to isolate in pure form as it exhibits a tendency for the reductive elimination of the indium fragment, which leads to the formation of the respective oxatetrasilacyclopentane.^15^ A similar behavior was reported to take place in the reaction of InCl_3_ with (Me_3_Si)_3_SiK.^46^

Tilley, Piers and others have shown that the Si–RE bond can be used for insertion chemistry with isonitriles, carbon monoxide or other small molecules.^9−11,21^ A maybe even more intriguing question is whether it is possible to do ligand exchange chemistry of silyl RE complexes that does **not** involve the Si–RE bond. To test this case, we reacted complex **2Y** with 2 equiv of CpNa·DME. Clean substitution of the two chlorides against cyclopentadienyl ligands was observed, and thus yttrocene ate-complex **5** (Scheme 2) could be isolated. The same compound is available via the reaction of Cp_2_YCl with oligosilanylene diide **1**, which can be considered analogous to our previously reported reactions of a dipotassium 1,4-oligosilanylene diide with Cp_3_Ln (Ln = Sm, Gd, Tb, Ho, and Tm) to form cyclic disilylated Cp_2_Ln complexes^13,17^ with a KCp unit acting as the leaving group.^13,17^

**Figure 2:**
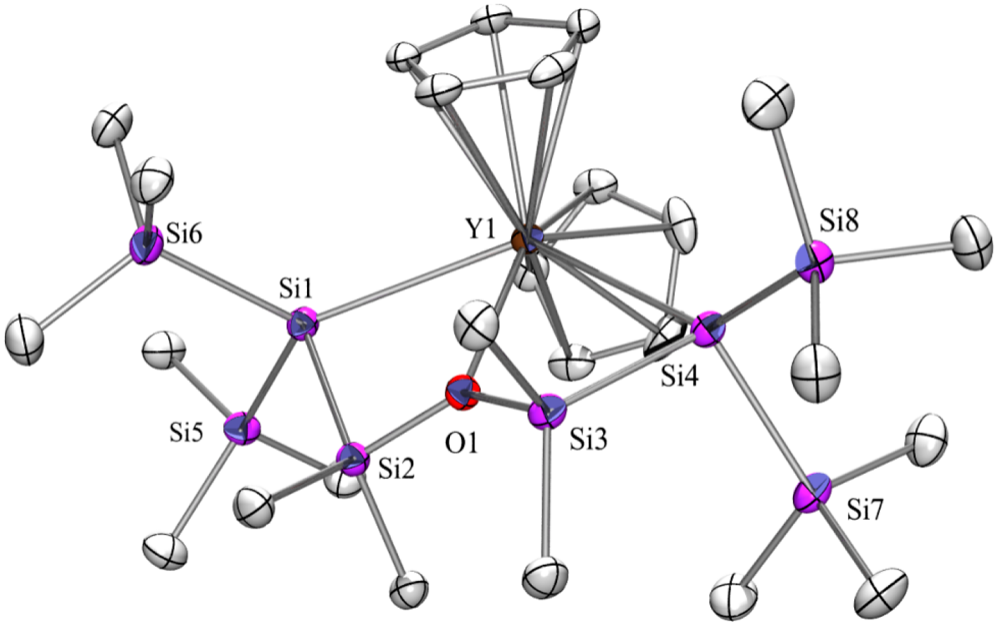
Molecular structure of **5** (thermal ellipsoid plot drawn at the 30% probability level). All hydrogen atoms are omitted for clarity (bond lengths in Å, angles in deg). Only the anionic part is shown. Y(1)–O(1) 2.484(2), Y(1)–C(4) 2.651(3), Y(1)–Si(1) 3.1315(9), Si(1)–Si(2) 2.3159(12), Si(1)–Si(6) 2.3449(12), Si(1)–Si(5) 2.3498(12), Si(2)–O(1) 1.705(2), Si(3)–O(1) 1.701(2), Si(2)–Si(1)–Si(6) 103.90(4), Si(2)–Si(1)–Si(5) 102.69(4), Si(6)–Si(1)–Y(1) 122.08(4), Si(5)–Si(1)–Y(1) 134.22(4), Si(3)–O(1)–Si(2) 136.25(13).

The ^29^Si NMR spectrum of **5** features signals at 23.7, −5.3, and −153.4 (d, ^1^*J*_Si–Y_ = 48 Hz) ppm. Compared to the signal at 37.7 ppm observed for **2Y**, the SiMe_2_O signal of **5** is shifted to higher field (23.7 ppm), suggesting that the Y–O interaction is weaker in **5**. The signal at −153.4 ppm is shifted slightly to lower field, and together with the somewhat larger ^1^*J*_Si–Y_ coupling constant of 48 Hz, this indicates a more covalent Si–Y interaction, still with a pronounced silanide character. Single-crystal XRD analysis of **5** (Figures 2 and S7, Table 1) reveals that the Si–Y distances of **5** (3.1315(9)/3.1459(9**)** Å) are significantly longer than those of **2Y** (3.064(2)/3.057(1) Å) and are by far the longest Si–Y bonds reported so far. The reason for this is likely of steric nature. The two Cp ligands of **5** unfold more steric demand than the two chlorides and the DME ligand of **2Y**. Nevertheless, the Y–Cp_centroid_ distances of **5** between 2.38 and 2.39 Å are quite typical. The respective values of Cp_3_Y^47,48^ are much more diverse, ranging from 2.37 to 2.50 Å. In accordance with the ^29^Si NMR signal for the SiMe_2_O unit the Y–O_SiMe2_ distance of **5** (2.484(2) Å) is larger than in **2Y** (2.422(2) Å).

## Conclusion

The chemistry of RE silyl complexes is receiving increased interest at the present time. Nevertheless, most reported studies so far have concentrated only on a few of the 17 RE elements. The reasons for this selective interest are not entirely clear but seem to be connected to facile isolation and characterization. For several of the RE elements, rigorous NMR characterization is difficult due to the elements’ inherent paramagnetic properties. Therefore, the importance of crystallographic characterization is eminent. However, it turns out the propensity for crystallization of complexes with the same silyl ligand and different RE elements can vary dramatically. All these reasons are likely responsible that we are still in a situation where there are **no** known examples for silyl complexes with several RE elements. In the current study, two of these blanks could be filled with the first examples of silyl complexes of lanthanum and praseodymium. In addition, we present the first structurally characterized example of a silyl dysprosium complex.

This was accomplished by reacting of a number of RE trichlorides with the oligosilanylene diide K(Me_3_Si)_2_SiSiMe_2_OSiMe_2_Si(SiMe_3_)_2_K (**1**). The thus formed products were of the type [(DME)_4_·K][(DME)·RE(Cl)_2_{Si(SiMe_3_)_2_SiMe_2_}_2_O] (RE = Y, La, Ce, Pr, Sm, Tb, Dy, and Er). The siloxane unit in the backbone of the ligand serves as an additional coordination site to the RE metal. Over the past years, we learned that one of the main decomposition pathways of silyl RE complexes involves the loss of weakly coordinated solvent molecules. The introduction of an additional Lewis basic site in the ligand and also the use of DME as potential bidentate ligand in the reaction, both contributed to enhance stability of our silyl RE ate complexes.

While the introduction of a ligand system that increases complex stability is a major achievement of this study; the other is that the obtained complexes [(DME)_4_·K][(DME)·RE(Cl)_2_{Si(SiMe_3_)_2_SiMe_2_}_2_O] contain two chloride ligands. These provide the principal possibility to serve as entry points to a whole class of complexes, where the chlorides are replaced by all sorts of other anionic ligands. The prototypical conversion of dichloro-yttrate complex **2Y** with 2 equiv of CpNa to yttrocene ate-complex **5** demonstrates the feasibility of this approach.

## Experimental Part

### General Remarks

All reactions involving air-sensitive compounds were carried out under an atmosphere of dry nitrogen or argon using either Schlenk techniques or a glovebox. All solvents were dried using a column based solvent purification system.^49^ Chemicals were obtained from different suppliers and used without further purification.

1,3-Bis[tris(trimethylsilyl)silyl]-1,1,3,3-tetramethyldisiloxane,^15^ 1,3-bis[potassiobis(trimethylsilyl)silyl]-1,1,3,3-tetramethyldisiloxane (**1**),^15^ Cp_2_YCl,^50^ and CpNa·DME^51^ were prepared following published procedures.

^1^H (300 MHz), ^13^C (75.4 MHz), and ^29^Si (59.3 MHz) NMR spectra were recorded on a Varian INOVA 300 spectrometer. Spectra are referenced to tetramethylsilane (TMS) for ^1^H, ^13^C, and ^29^Si. If not otherwise noted, all samples were measured in C_6_D_6_. To compensate for the low isotopic abundance of ^29^Si, either the DEPT or INEPT pulse sequences were used for the amplification of the signal.^52−54^

Due to the difficulties in isolating the DME adducts **2RE** in the solid state, no satisfactory elemental analyses could be obtained.

For single-crystal X-ray diffraction structure analyses, crystals were mounted onto the tip of glass fibers, and data collection was performed with a BRUKER-AXS SMART APEX CCD diffractometer using graphite-monochromated Mo Kα radiation (0.71073 Å). The data were reduced to *F*^2^_o_ and corrected for absorption effects with SAINT^55^ and SADABS,^56,57^ respectively. Structures were solved by direct methods and refined by full-matrix least-squares method (SHELXL97).^58^ If not otherwise noted, all non-hydrogen atoms were refined with anisotropic displacement parameters. All hydrogen atoms were located in calculated positions to correspond to standard bond lengths and angles. All diagrams were drawn with 30% probability thermal ellipsoids and all hydrogen atoms were omitted for clarity. Crystallographic data for the structures of compounds **2Y**, **2Ce**, **2Pr**, **2Sm**, **2Tb**, **2Tb**, and **5** reported in this paper are deposited with the Cambridge Crystallographic Data Center as supplementary publication no. CCDC 2071459 (**2Y**), 2071465 (**2Ce**), 2071460 (**2Pr**), 2071457 (**2Sm**), 2071467 (**2Tb**), 2071462 (**2Dy**), and 2071466 (**5**). Copies of data can be obtained free of charge at: http://www.ccdc.cam.ac.uk/products/csd/request/. Figures of solid-state molecular structures were generated using Ortep-3 as implemented in WINGX^59^ and rendered using POV-Ray 3.6.^60^

### General Procedure for the Preparation of [(DME)_4_·K][(DME)·RE(Cl)_2_{Si(SiMe_3_)_2_SiMe_2_}_2_O] (**2RE**)

1,3-Bis[potassiobis(trimethylsilyl)silyl]-1,1,3,3-tetramethyldisiloxane (**1**) was prepared starting from 1,3-bis[tris(trimethylsilyl)silyl]-1,1,3,3-tetramethyldisiloxane (1.00 equiv) and potassium *tert*-butoxide (2.06 equiv) in DME (3 mL). After 19 h at room temperature, full conversion was detected by ^29^Si NMR spectroscopic analysis. Volatiles were removed under reduced pressure, and the residue was dissolved in DME (1.5 mL). The bright yellow solution was added dropwise within 2 min to a slurry of the respective RE chloride RECl_3_ (1.06 equiv) in DME (0.5 mL). The mixture was stirred for a specified time, before the formed precipitate was removed through centrifugation and filtration. Crystalline products were obtained from pentane/DME solutions at −50 °C. Isolation of these products has to be accomplished with utmost care as the removal of DME in vacuum results in decomposition.

### [(DME)_4_·K][(DME)·Y(Cl)_2_{Si(SiMe_3_)_2_SiMe_2_}_2_O] (**2Y**)

Following the general procedure, we used 1,3-bis[tris(trimethylsilyl)silyl]-1,1,3,3-tetramethyldisiloxane (150 mg, 0.24 mmol), potassium *tert*-butoxide (56 mg, 0.50 mmol), and yttrium trichloride (52 mg, 0.27 mmol) in DME (2 mL). The mixture was stirred for 90 min. Colorless crystals of **2Y** (209 mg, 77%) were obtained. NMR (δ in ppm, THF-*d*_8_): ^1^H: 3.43 (s, coord. DME), 3.27 (s, coord. DME), 0.50 (s, 12H), 0.14 (s, 36H). ^13^C: 72.8, 59.0, 9.9, 6.3. ^29^Si (D_2_O-capillary/DME): 37.7 (d, ^2^*J*_Si–Y_ = 3 Hz, SiMe_2_), −6.5 (s, SiMe_3_), −161.6 (d, ^1^*J*_Si–Y_ = 38 Hz, Si*_q_*).

### [(DME)_4_·K][(DME)·La(Cl)_2_{Si(SiMe_3_)_2_SiMe_2_}_2_O] (**2La**)

Following the general procedure, we used 1,3-bis[tris(trimethylsilyl)silyl]-1,1,3,3-tetramethyldisiloxane (151 mg, 0.24 mmol), potassium *tert*-butoxide (55 mg, 0.49 mmol), and lanthanum trichloride (64 mg, 0.26 mmol) in DME (2 mL). The mixture was stirred for 185 min, and colorless crystals of **2La** (200 mg, 71%) were obtained from a pentane/DME solution at −50 °C. NMR (δ in ppm, D_2_O-capillary/DME): ^1^H: 0.48 (s, 12H), 0.12 (s, 36H). ^13^C: 9.2, 5.0. ^29^Si: 36.9 (s, SiMe_2_), −5.7 (s, SiMe_3_), n.d. (Si*_q_*).

### [(DME)_4_·K][(DME)·Ce(Cl)_2_{Si(SiMe_3_)_2_SiMe_2_}_2_O] (**2Ce**)

Following the general procedure, we used 1,3-bis[tris(trimethylsilyl)silyl]-1,1,3,3-tetramethyldisiloxane (151 mg, 0.24 mmol), potassium *tert*-butoxide (55 mg, 0.49 mmol), and cerium trichloride (66 mg, 0.27 mmol) in DME (2 mL). The mixture was stirred for 90 min, and light yellow crystals of **2Ce** (260 mg, 92%) were obtained from a pentane/DME solution at −60 °C. NMR (δ in ppm, THF-*d*_8_/DME/pentane): ^1^H: 3.46 (s, coord. DME), 3.28 (s, coord. DME), 1.16 (s, 12H), −0.32 (s, 36H). ^1^^3^C: 72.8 (coord. DME), 58.9 (coord. DME), 9.5 (SiMe_3_), 8.3 (SiMe_2_).

### [(DME)_4_·K][(DME)·Pr(Cl)_2_{Si(SiMe_3_)_2_SiMe_2_}_2_O] (**2Pr**)

Following the general procedure, we used 1,3-bis[tris(trimethylsilyl)silyl]-1,1,3,3-tetramethyldisiloxane (150 mg, 0.24 mmol), potassium *tert*-butoxide (56 mg, 0.50 mmol), and praseodymium trichloride (64 mg, 0.26 mmol) in DME (2 mL). The mixture was stirred for 150 min, and leek green crystals of **2Pr** (152 mg, 54%) were obtained. NMR (δ in ppm, (D_2_O-capillary/DME): ^1^H: broad peaks around 3.0 and 0.0 ppm. No ^13^C or ^29^Si NMR spectra could be obtained for the paramagnetic **2Pr**.

### [(DME)_4_·K][(DME)·Sm(Cl)_2_{Si(SiMe_3_)_2_SiMe_2_}_2_O] (**2Sm**)

Following the general procedure, we used 1,3-bis[tris(trimethylsilyl)silyl]-1,1,3,3-tetramethyldisiloxane (151 mg, 0.24 mmol), potassium *tert*-butoxide (55 mg, 0.49 mmol), and samarium trichloride (67 mg, 0.26 mmol) in DME (2 mL). The mixture was stirred for 90 min, and deep purple crystals of **2Sm** (180 mg, 63%) were obtained. NMR (δ in ppm, D_2_O-capillary/DME): ^1^H: 0.30 (s, 36H), 0.13 (s, 12H). ^13^C: 9.9 (SiMe_3_), 5.2 (SiMe_2_). ^29^Si: 42.9 (s, SiMe_2_), −9.0 (s, SiMe_3_), n.d. (Si*_q_*).

### [(DME)_4_·K][(DME)·Tb(Cl)_2_{Si(SiMe_3_)_2_SiMe_2_}_2_O] (**2Tb**)

Following the general procedure, we used 1,3-bis[tris(trimethylsilyl)silyl]-1,1,3,3-tetramethyldisiloxane (150 mg, 0.24 mmol), potassium *tert*-butoxide (55 mg, 0.49 mmol), and terbium trichloride (69 mg, 0.26 mmol) in DME (2 mL). The mixture was stirred for 105 min, and colorless crystals of **2Tb** (175 mg, 61%) were obtained. NMR (δ in ppm, THF-*d*_8_): ^1^H: 22.89 (bs), 3.92 (bs). No ^13^C or ^29^Si NMR spectra could be obtained for the paramagnetic **2Tb**.

### [(DME)_4_·K][(DME)·Dy(Cl)_2_{Si(SiMe_3_)_2_SiMe_2_}_2_O] (**2Dy**)

Following the general procedure, we used 1,3-bis[tris(trimethylsilyl)silyl]-1,1,3,3-tetramethyldisiloxane (151 mg, 0.24 mmol), potassium *tert*-butoxide (56 mg, 0.50 mmol), and dysprosium trichloride (69 mg, 0.26 mmol) in DME (2 mL). The mixture was stirred for 165 min, and yellow crystals of **2Dy** (150 mg, 52%) were obtained. NMR (δ in ppm, (D_2_O-capillary/DME): ^1^H: two broad peaks around 23 ppm. No ^13^C or ^29^Si NMR spectra could be obtained for the paramagnetic **2Dy**.

### [(DME)_4_·K][(DME)·Er(Cl)_2_{Si(SiMe_3_)_2_SiMe_2_}_2_O] (**2Er**)

Following the general procedure, we used 1,3-bis[tris(trimethylsilyl)silyl]-1,1,3,3-tetramethyldisiloxane (150 mg, 0.24 mmol), potassium *tert*-butoxide (56 mg, 0.50 mmol), and erbium trichloride (69 mg, 0.25 mmol) in DME (2 mL). The mixture was stirred for 165 min, and slightly pinkish crystals of **2Er** (70 mg, 24%) were obtained. NMR (δ in ppm, (D_2_O-capillary/DME): ^1^H: 29.64 (coord.-DME, bs, 4H), 16.43 (coord.-DME, bs, 4H), 15.34 (bs, 36H), 7.31 (bs, 12H). No ^13^C or ^29^Si NMR spectra could be obtained for paramagnetic **2Er**. The compound is highly light-sensitive, as clear solutions turned immediately cloudy upon exposure to light. Moreover, crystals of **2Er** are very soluble in minimal amounts of solvent, which prevented its XRD characterization.

### Cp_2_Zr{Si(SiMe_3_)_2_SiMe_2_}_2_O] (**3**)

A solution of compound **2Y** (freshly prepared from 1,3-bis[tris(trimethylsilyl)silyl]-1,1,3,3-tetramethyldisiloxane (150 mg, 0.24 mmol), potassium *tert*-butoxide (56 mg, 0.50 mmol), and yttrium trichloride (50 mg, 0.26 mmol)) in DME (2 mL) was added dropwise to bis(cyclopentadienyl)zirconium dichloride (80 mg, 0.27 mmol) and stirred at room temperature for 100 min. Volatiles were removed, and the residue was extracted with pentane (2 × 2 mL) to give a blood red solution. Pentane was removed in vacuo to give **3** (90 mg, 54% over three steps) as a blood red powder. Observed NMR data were found to be in accordance with reported literature values.^16^

### [(DME)_4_·K][In(Cl)_2_{Si(SiMe_3_)_2_SiMe_2_}_2_O] (**4**)

A solution of compound **2Y** (freshly prepared from tetrakis(trimethylsilyl)silyl]-1,1,3,3-tetramethyldisiloxane (150 mg, 0.24 mmol), potassium *tert*-butoxide (55 mg, 0.49 mmol), and yttrium trichloride (51 mg, 0.26 mmol)) in DME (1.5 mL) was added dropwise to indium trichloride (43 mg, 0.27 mmol) and stirred at room temperature under the exclusion of light for 135 min. Precipitates were removed through centrifugation and filtration, and the slightly yellow solution was subjected to NMR analysis indicating the formation of **4**. Upon storage at room temperature, colorless crystals of **4** (120 mg, 47%) were obtained. NMR (δ in ppm): ^1^H (THF-*d*_8_): 0.52 (s, 12H), 0.33 (s, 36H). ^13^C (THF-*d*_8_): 8.7, 3.5. ^29^Si (D_2_O capillary/DME): 13.5 (s, SiMe_2_), −6.4 (s, SiMe_3_), −131.8 (Si*_q_*). Complete removal of solvents from a solution of **4** was found to cause decomposition, which is evidenced by the precipitation of a metallic, greyish solid and the formation of several decomposition products of the silyl ligand. The compound is highly light-sensitive, as a clear solution forms a metallic powder upon exposure to light.

### [(DME)_4_·K][Cp_2_Y{Si(SiMe_3_)_2_SiMe_2_}_2_O] (**5**)

#### Method A

A mixture of compound **2Y** (freshly prepared from 1,3-bis[tris(trimethylsilyl)silyl]-1,1,3,3-tetramethyldisiloxane (151 mg, 0.25 mmol), potassium *tert*-butoxide (57 mg, 0.51 mmol), and yttrium trichloride (53 mg, 0.27 mmol) in DME (2 mL)) was added dropwise to a solution of cyclopentadienyl sodium·DME (91 mg, 0.51 mmol) in DME (0.5 mL). After stirring for 4 h, the insoluble parts were removed by centrifugation and filtration, and the bright yellow solution was subjected to NMR analysis, confirming the formation of **5** (180 mg, 68%).

#### Method B

Following the general procedure, we used 1,3-bis[tris(trimethylsilyl)silyl]-1,1,3,3-tetramethyldisiloxane (89 mg, 0.14 mmol), potassium *tert*-butoxide (33 mg, 0.29 mmol), and bis(cyclopentadienyl)yttrium chloride (37 mg, 0.15 mmol) in DME (2 mL)). The mixture was stirred for 20 h, before the precipitate was removed through centrifugation and filtration. Slightly yellow crystals of **5** (130 mg, 91%) were obtained from a pentane/DME solution at −50 °C. Mp: 283 °C (decomp.).

NMR (δ in ppm, THF-*d*_8_): ^1^H: 6.19 (s, 10H), 3.44 (s, 12H), 3.28 (s, 18H), 0.26 (s, 12H), 0.21 (s, 36H). ^13^C: 107.3, 71.7, 57.9, 9.6, 5.8. ^29^Si (D_2_O-capillary/DME): 23.7 (s, SiMe_2_), −5.3 (s, SiMe_3_), −153.4 (d,^1^*J*_Si–Y_ = 48 Hz, Si*_q_*).

## Supporting Information Available

Tabulated crystallographic data and ORTEP plots with selected structural data of for compounds **2Y**, **2Ce**, **2Pr**, **2Sm**, **2Tb**, **2Dy**, and **5**. NMR spectra (^1^H, ^13^C, and ^29^Si) for compounds **2Y**, **2La**, **2Ce**, **2Pr**, **2Sm**, **2Tb**, **2Dy**, **2Er**, **4**, and **5** (PDF)

## Accession Codes

CCDC 2071457, 2071459, 2071460, 2071462, and 2071465–2071467 contain the supplementary crystallographic data for this paper. These data can be obtained free of charge via www.ccdc.cam.ac.uk/data_request/cif, or by emailing data_request@ccdc.cam.ac.uk, or by contacting The Cambridge Crystallographic Data Centre, 12 Union Road, Cambridge CB2 1EZ, UK; fax: +44 1223 336033.
