# Supplementary material for: Rare-Earth–Silyl ate-Complexes Opening a Door to Selective Manipulations
Source: Inorg Chem. 2021 May 25;60(11):8218–26. doi: 10.1021/acs.inorgchem.1c00904 (PMC8188526; doi:10.1021/acs.inorgchem.1c00904)
Supplement: Supplementary file 1 — ic1c00904_si_001.pdf [file ic1c00904_si_001.pdf]

Supporting Information for

Rare Earth-Silyl ate-Complexes Opening a Door to Selective Manipulations

Alexander Pöcheim, Christoph Marschner,* and Judith Baumgartner*

Institut für Anorganische Chemie, Technische Universität Graz, Stremayrgasse 9, 8010 Graz, Austria

Contents

1. Crystallographic tables of compounds **2Y**, **2Ce**, **2Pr**, **2Sm**, **2Tb**, **2Dy**, and **5** **S1**
2. Figures of the solid state structures of **2Y**, **2Ce**, **2Pr**, **2Sm**, **2Tb**, **2Dy**, and **5** **S3**
3. NMR spectra **S10**

^{1}H, ^{13}C and ^{29}Si Spectra **2Y** **S10**

^{1}H, ^{13}C and ^{29}Si Spectra **2La** **S11**

^{1}H and ^{13}C Spectra **2Ce** **S13**

^{1}H Spectrum **2Pr** **S14**

^{1}H, ^{13}C and ^{29}Si Spectra **2Sm** **S14**

^{1}H Spectrum **2Tb** **S16**

^{1}H Spectrum **2Dy** **S16**

^{1}H Spectrum **2Er** **S17**

^{1}H, ^{13}C and ^{29}Si Spectra **4** **S17**

^{1}H, ^{13}C and ^{29}Si Spectra **5** **S19**

1. Crystallographic Tables

Table S1. Crystallographic data for compounds **2Y**, **2Ce**, **2Pr**, and **2Sm**.

	**2Y**	**2Ce**	**2Pr**	**2Sm**
Empirical formula	$C_{36}H_{98}Cl_2KO_{11}Si_8Y$	$C_{36}H_{98}CeCl_2KO_{11}Si_8$	$C_{72}H_{196}Cl_4K_2O_{22}Pr_2Si_{16}$	$C_{36}H_{98}Cl_2KO_{11}Si_8Sm$
M_w	1130.77	1181.98	2365.55	1192.21
Temperature [K]	100(2)	200(2)	100(2)	100(2)
Size [mm]	0.38×0.32×0.07	0.26×0.24×0.11	0.32×0.26×0.20	0.36×0.24×0.14
Crystal system	triclinic	triclinic	monoclinic	triclinic
Space group	P-1	P-1	P2(1)/c	P-1
a [Å]	14.527(3)	14.578(4)	14.785(3)	14.488(4)
b [Å]	14.546(3)	14.660(4)	31.767(6)	14.497(4)
c [Å]	15.748(3)	16.118(5)	28.625(6)	15.852(5)
α [°]	85.328(4)	94.370(4)	90	89.236(5)
β [°]	84.184(4)	90.628(5)	102.968(4)	84.795(5)
γ [°]	77.282(4)	103.994(4)	90	75.907(4)
V [$Å^3$]	3223(2)	3331(2)	13102(4)	3216(2)
Z	2	2	4	2
ρ_{calc} [gcm^{-3}]	1.165	1.178	1.199	1.231
Absorption coefficient [mm^{-1}]	1.242	1.010	1.076	1.251
F(000)	1212	1250	5008	1258
θ range	1.44<θ<26.35	1.44<θ<26.37	0.97<θ<25.00	1.45<θ<26.33
Reflections collected/unique	16999/11992	22159/12931	94382/23066	25241/12835
Completeness to θ [%]	91.2	94.9	100	98.1
Data/restraints/parameters	11992/0/558	12931/0/558	23066/6/1115	12835/0/701
Goodness of fit on F^2	0.96	1.13	1.39	1.02
Final R indices [I>2σ(I)]	R1=0.065, *w*R2=0.153	R1=0.067, *w*R2=0.155	R1=0.103, *w*R2=0.198	R1=0.038, *w*R2=0.093
R indices (all data)	R1=0.099, *w*R2=0.167	R1=0.080, *w*R2=0.163	R1=0.112, *w*R2=0.201	R1=0.042, *w*R2=0.095
Largest diff. Peak/hole [e^-/ $Å^3$]	2.04/−0.92	1.39/−0.72	1.47/−1.81	1.66/−0.88

Table S2. Crystallographic data for compounds **2Tb**, **2Dy**, and **5**.

	**2Tb**	**2Dy**	**5**
Empirical formula	$C_{36}H_{98}Cl_2KO_{11}Si_8Tb$	$C_{36}H_{96}Cl_2DyKO_{11}Si_8$	$C_{42}H_{98}KO_9Si_8Y$
M_w	1200.78	1202.35	1099.93
Temperature [K]	100(2)	150(2)	100(2)
Size [mm]	0.32×0.28×0.14	0.26×0.20×0.12	0.30×0.26×0.18
Crystal system	triclinic	triclinic	monoclinic
Space group	P-1	P-1	P2(1)/c
a [Å]	14.532(7)	14.598(5)	13.727(3)
b [Å]	14.562(7)	14.654(5)	30.203(6)
c [Å]	15.861(8)	15.995(6)	15.537(3)
α [°]	88.930(8)	89.254(6)	90
β [°]	84.602(8)	84.513(6)	106.016(3)
γ [°]	75.924(8)	75.808(6)	90
V [$Å^3$]	3241(3)	3302(2)	6192(2)
Z	2	2	4
ρ_{calc} [gcm^{-3}]	1.230	1.209	1.180
Absorption coefficient [mm^{-1}]	1.427	1.461	1.206
F(000)	1264	1262	2368
θ range	1.44<θ<26.37	1.43<θ<25.00	1.35<θ<26.36
Reflections collected/unique	25338/12997	23073/11486	48897/12541
Completeness to θ [%]	98.1	98.6	99.2
Data/restraints/parameters	12997/0/710	11486/30/632	12541/0/572
Goodness of fit on F^2	1.04	1.23	1.028
Final R indices [I>2σ(I)]	R1=0.063, *w*R2=0.150	R1=0.109, *w*R2=0.253	R1=0.045, *w*R2=0.098
R indices (all data)	R1=0.078, *w*R2=0.158	R1=0.111, *w*R2=0.254	R1=0.066, *w*R2=0.106
Largest diff. Peak/hole [e^-/ $Å^3$]	5.59/−1.41	8.13/−3.20	1.08/−0.84

2. Figures of the solid state structures of 2Y, 2Ce, 2Pr, 2Sm, 2Tb, 2Dy, and 5

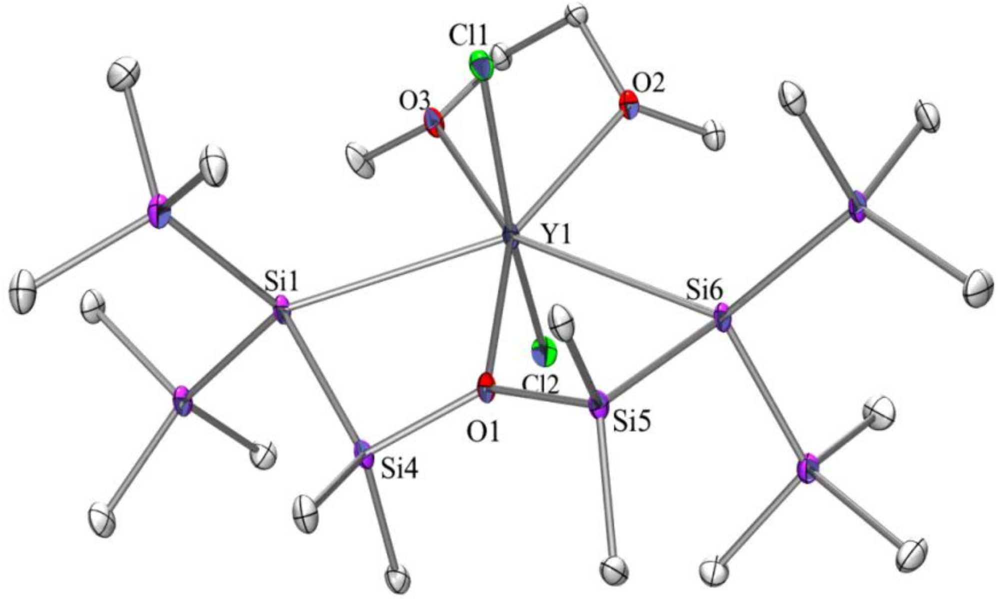

Figure S1. Molecular structure of **2Y** (thermal ellipsoid plot drawn at the 30% probability level). All hydrogen atoms are omitted for clarity (bond lengths in Å, angles in deg). Only the anionic part is shown. Y(1)-O(1) 2.422(3), Y(1)-O(2) 2.443(3), Y(1)-Cl(1) 2.5840(13), Y(1)-Cl(2) 2.5854(13), Y(1)-Si(1) 3.0575(13), Y(1)-Si(6) 3.0641(13), O(1)-Si(5) 1.707(3), O(1)-Si(4) 1.710(3), O(2)-C(18) 1.437(5), Si(1)-Si(4) 2.3072(18), Si(2)-C(1) 1.885(6), Si(5)-Si(6) 2.3118(18), O(2)-Y(1)-O(3) 66.30(10), Cl(1)-Y(1)-Cl(2) 165.86(4), Si(1)-Y(1)-Si(6) 133.07(4), Si(5)-O(1)-Si(4) 136.0(2), Si(4)-Si(1)-Y(1) 78.46(5), Si(5)-Si(6)-Y(1) 78.57(5).

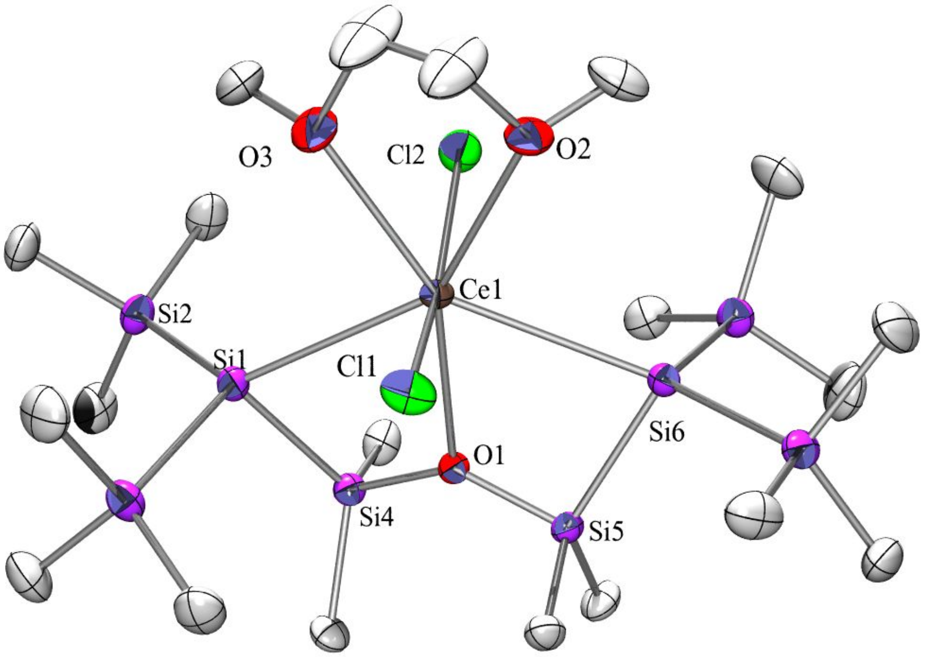

Figure S2. Molecular structure of **2Ce** (thermal ellipsoid plot drawn at the 30% probability level). All hydrogen atoms are omitted for clarity (bond lengths in Å, angles in deg). Only the anionic part shown. Ce(1)-O(1) 2.574(3), Ce(1)-O(2) 2.613(4), Ce(1)-O(3) 2.632(4), Ce(1)-Cl(2) 2.6881(16), Ce(1)-Cl(1) 2.7089(16), Ce(1)-Si(1) 3.1442(16), Ce(1)-Si(6) 3.1593(16), Si(4)-O(1) 1.705(3), Si(1)-Si(4) 2.318(2), Si(5)-O(1) 1.696(3), Si(5)-Si(6) 2.315(2), O(2)-Ce(1)-O(3) 63.53(15), Cl(2)-Ce(1)-Cl(1) 162.29(5), Si(5)-Ce(1)-Si(2) 129.49(4).

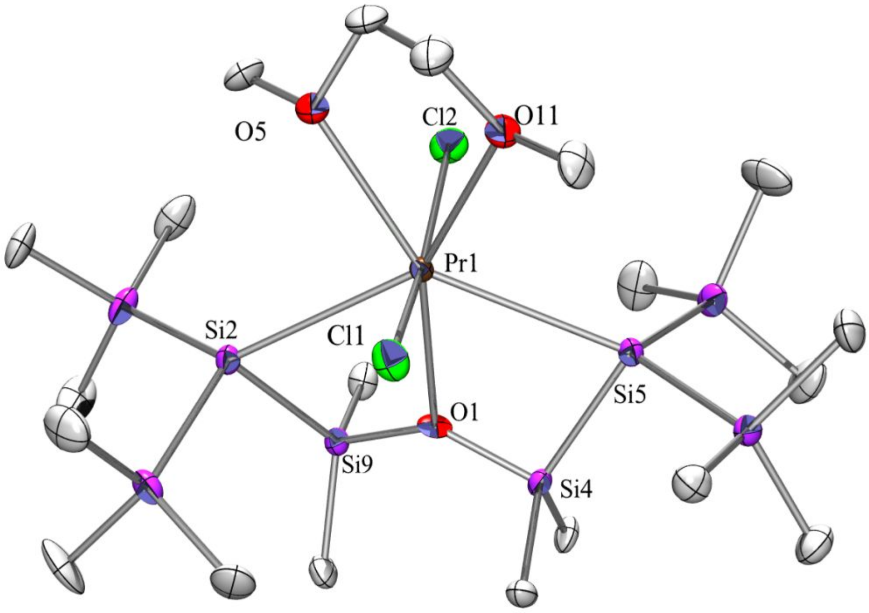

Figure S3. Molecular structure of **2Pr** (thermal ellipsoid plot drawn at the 30% probability level). All hydrogen atoms are omitted for clarity (bond lengths in Å, angles in deg). Of two independent complex entities only the anionic part of one is shown. Pr(1)-O(1) 2.508(5), Pr(1)-O(11) 2.616(6), Pr(1)-O(5) 2.632(6), Pr(1)-Cl(2) 2.681(3), Pr(1)-Cl(1) 2.684(2), Pr(1)-Si(5) 3.159(2), Pr(1)-Si(2) 3.167(2), Si(9)-O(1) 1.738(6), Si(9)-C(3) 1.865(9), Si(9)-Si(2) 2.318(3), Si(4)-O(1) 1.698(6), Si(4)-Si(5) 2.319(3), O(11)-Pr(1)-O(5) 64.0(2), Cl(2)-Pr(1)-Cl(1) 165.02(9), Si(5)-Pr(1)-Si(2) 130.49(7), O(1)-Si(4)-Si(5) 99.9(2), Si(4)-O(1)-Si(9) 136.6(3).

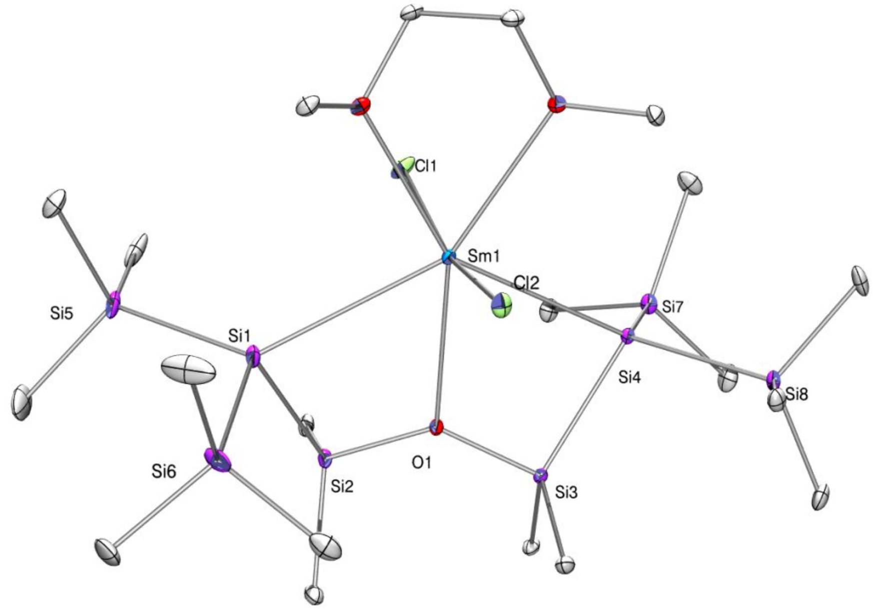

Figure S4. Molecular structure of **2Sm** (thermal ellipsoid plot drawn at the 30% probability level). All hydrogen atoms are omitted for clarity (bond lengths in Å, angles in deg). Only the anionic part shown. Sm(1)-O(1) 2.501(2), Sm(1)-O(2) 2.528(2), Sm(1)-Cl(2) 2.6390(10), Sm(1)-Cl(1) 2.6491(10), Sm(1)-Si(1) 3.0891(11), Sm(1)-Si(4) 3.0987(10), Si(1)-Si(2) 2.3103(13), Si(1)-Si(5) 2.3240(16), Si(1)-Si(6) 2.3348(17), Si(2)-O(1) 1.705(2), Si(3)-O(1) 1.696(2), Si(3)-C(6) 1.865(3), Si(3)-Si(4) 2.3161(13), Si(4)-Si(8) 2.3286(13), Si(4)-Si(7) 2.3332(14), Cl(2)-Sm(1)-Cl(1) 164.72(3), Si(1)-

Sm(1)-Si(4) 131.63(3), Si(2)-Si(1)-Si(5) 108.52(5), Si(2)-Si(1)-Si(6) 105.97(6), Si(5)-Si(1)-Si(6) 108.38(6).

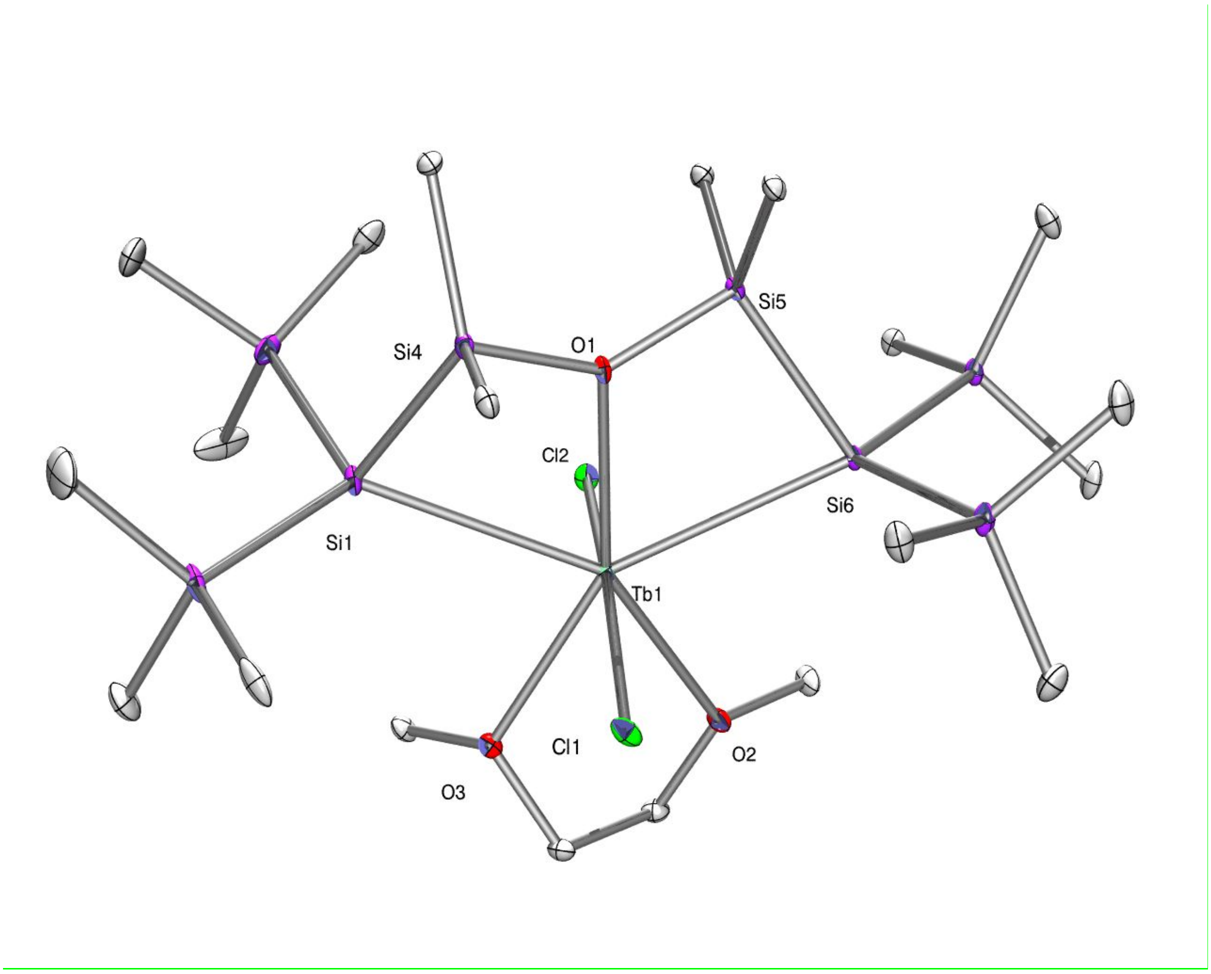

Figure S5. Molecular structure of **2Tb** (thermal ellipsoid plot drawn at the 30% probability level). All hydrogen atoms are omitted for clarity (bond lengths in Å, angles in deg). Only the anionic part shown. Tb(1)-O(1) 2.470(4), Tb(1)-O(3) 2.501(4), Tb(1)-O(2) 2.566(4), Tb(1)-Cl(2) 2.6103(17), Tb(1)-Cl(1) 2.6213(17), Tb(1)-Si(1) 3.0733(18), Tb(1)-Si(6) 3.0814(17), Si(1)-Si(4) 2.318(2), Si(2)-C(1) 1.876(9), Si(4)-O(1) 1.711(4), Si(5)-O(1) 1.703(4), Si(5)-Si(6) 2.323(2), O(2)-C(18) 1.435(7), O(3)-Tb(1)-O(2) 65.69(13), Cl(2)-Tb(1)-Cl(1) 165.57(5), Si(1)-Tb(1)-Si(6) 132.70(5), Si(4)-Si(1)-Tb(1) 78.90(6), Si(5)-Si(6)-Tb(1) 78.73(6), Si(5)-O(1)-Si(4) 137.2(2).

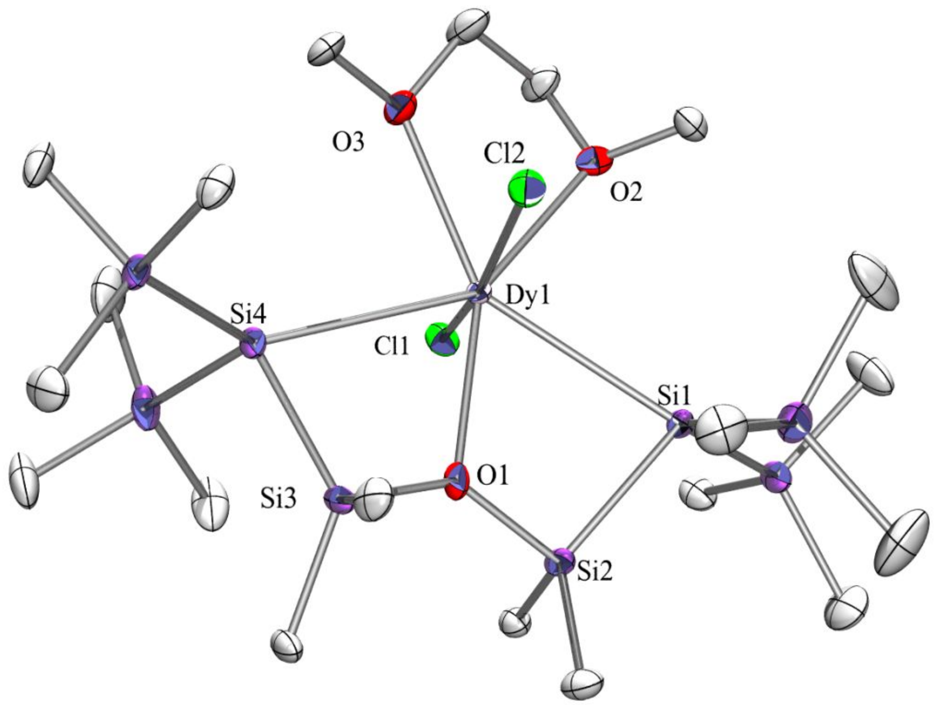

Figure S6. Molecular structure of **2Dy** (thermal ellipsoid plot drawn at the 30% probability level). All hydrogen atoms are omitted for clarity (bond lengths in Å, angles in deg). Only the anionic part shown. Dy(1)-O(1) 2.454(9), Dy(1)-O(2) 2.517(9), Dy(1)-O(3) 2.549(9), Dy(1)-Cl(2) 2.596(3), Dy(1)-Cl(1) 2.603(3), Dy(1)-Si(4) 3.077(3), Dy(1)-Si(1) 3.077(3), O(1)-Si(3) 1.700(8), O(1)-Si(2) 1.726(8), Si(1)-Si(2) 2.319(5), Si(2)-C(1) 1.873(15), Si(3)-Si(4) 2.327(5), O(2)-Dy(1)-O(3) 65.4(3), Cl(2)-Dy(1)-Cl(1) 165.79(11), Si(4)-Dy(1)-Si(1) 132.94(9), Si(3)-O(1)-Si(2) 136.6(6).

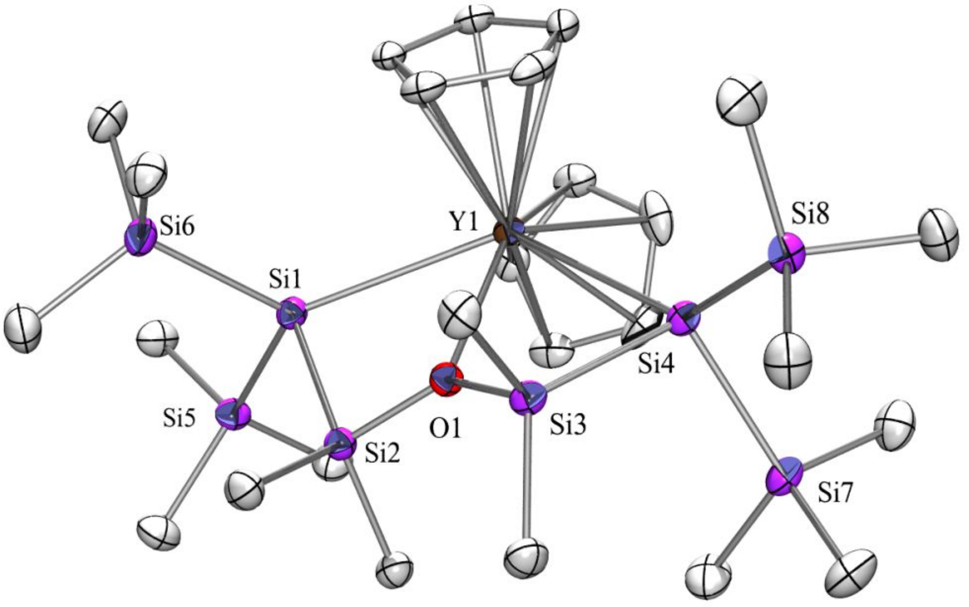

Figure S7. Molecular structure of **5** (thermal ellipsoid plot drawn at the 30% probability level). All hydrogen atoms are omitted for clarity (bond lengths in Å, angles in deg). Only the anionic part shown. Y(1)-O(1) 2.484(2), Y(1)-C(4) 2.651(3), Y(1)-Si(1) 3.1315(9), Si(1)-Si(2) 2.3159(12), Si(1)-Si(6) 2.3449(12), Si(1)-Si(5) 2.3498(12), Si(2)-O(1) 1.705(2), Si(3)-O(1) 1.701(2), Si(2)-Si(1)-Si(6) 103.90(4), Si(2)-Si(1)-Si(5) 102.69(4), Si(6)-Si(1)-Y(1) 122.08(4), Si(5)-Si(1)-Y(1) 134.22(4), Si(3)-O(1)-Si(2) 136.25(13).

3. ^{1}H, ^{13}C, and ^{29}Si NMR spectra

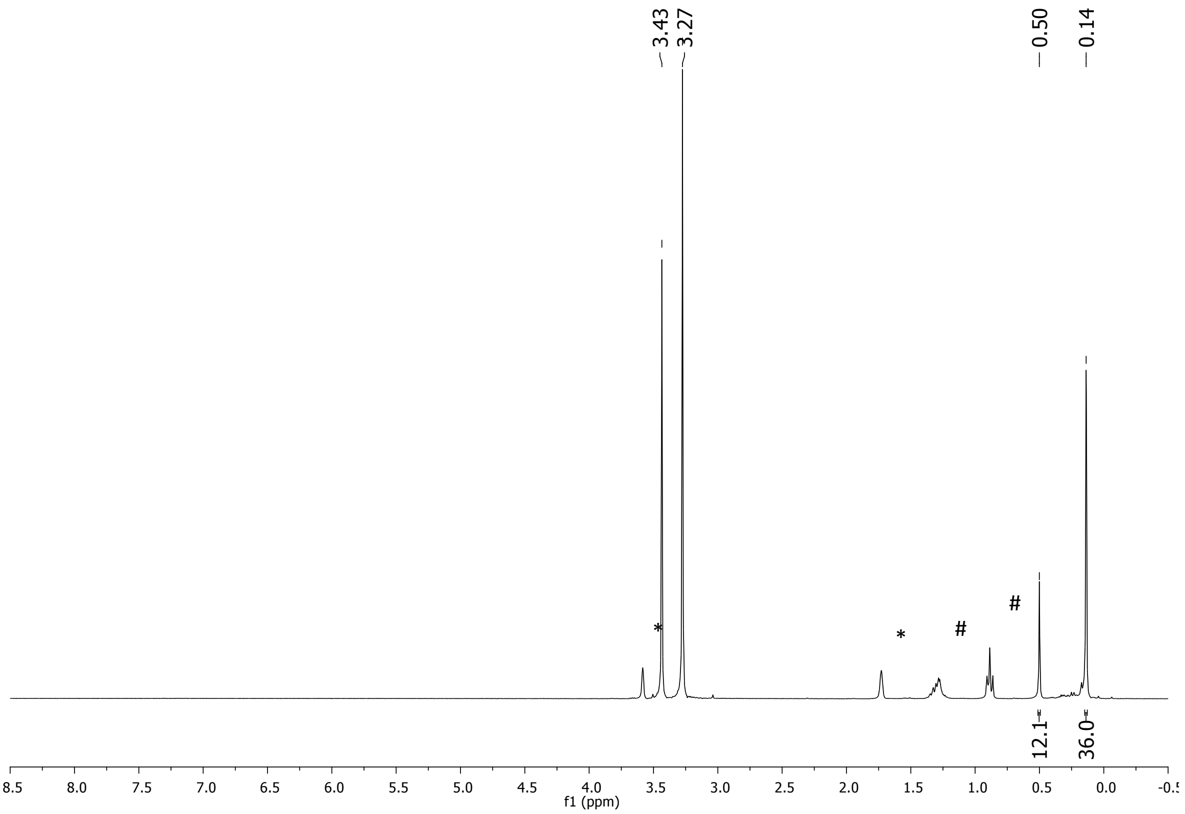

Figure S8. *^{1}H NMR spectrum of **2Y** in d^8-THF. Solvent related signals marked as following: d^8-THF (*), pentane (#).*

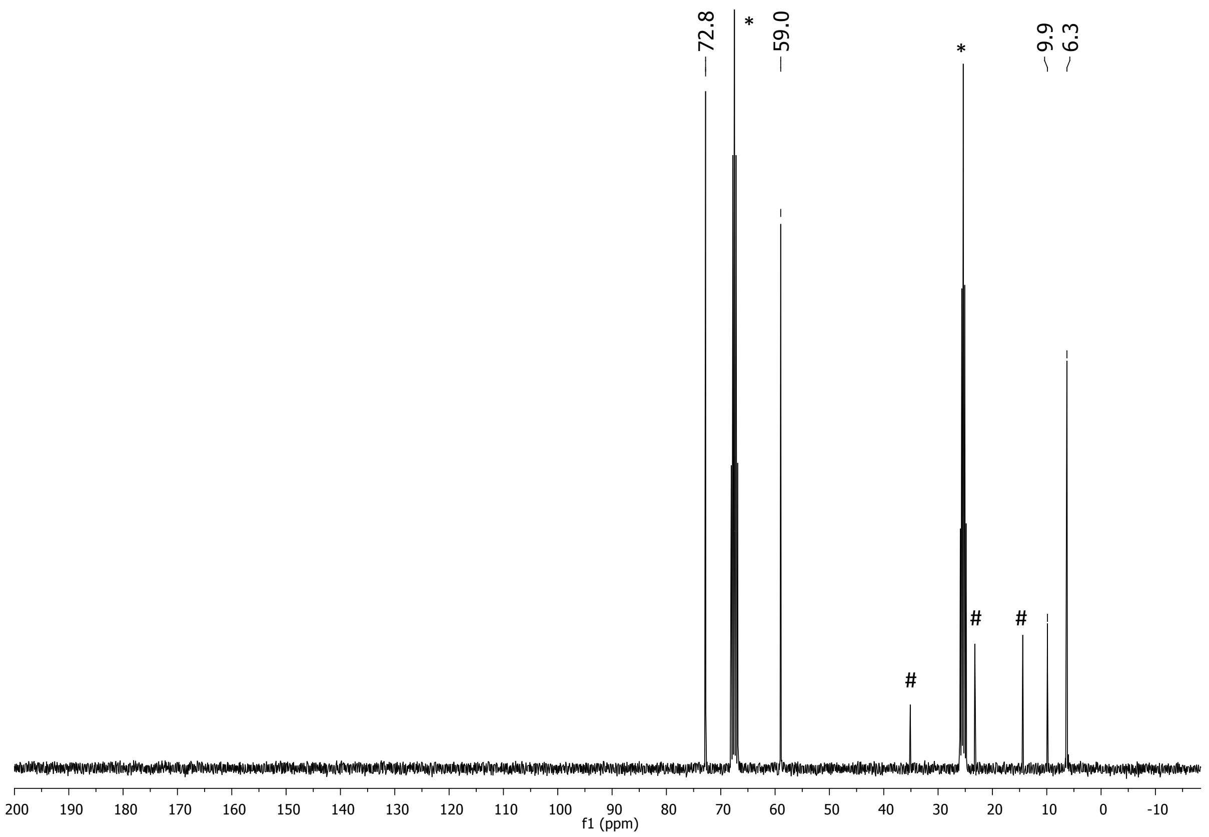

Figure S9. ^{13}C *NMR spectrum of* ***2Y*** *in* d^8*-THF. Solvent related signals marked as following:* d^8*-THF (*), pentane (#).*

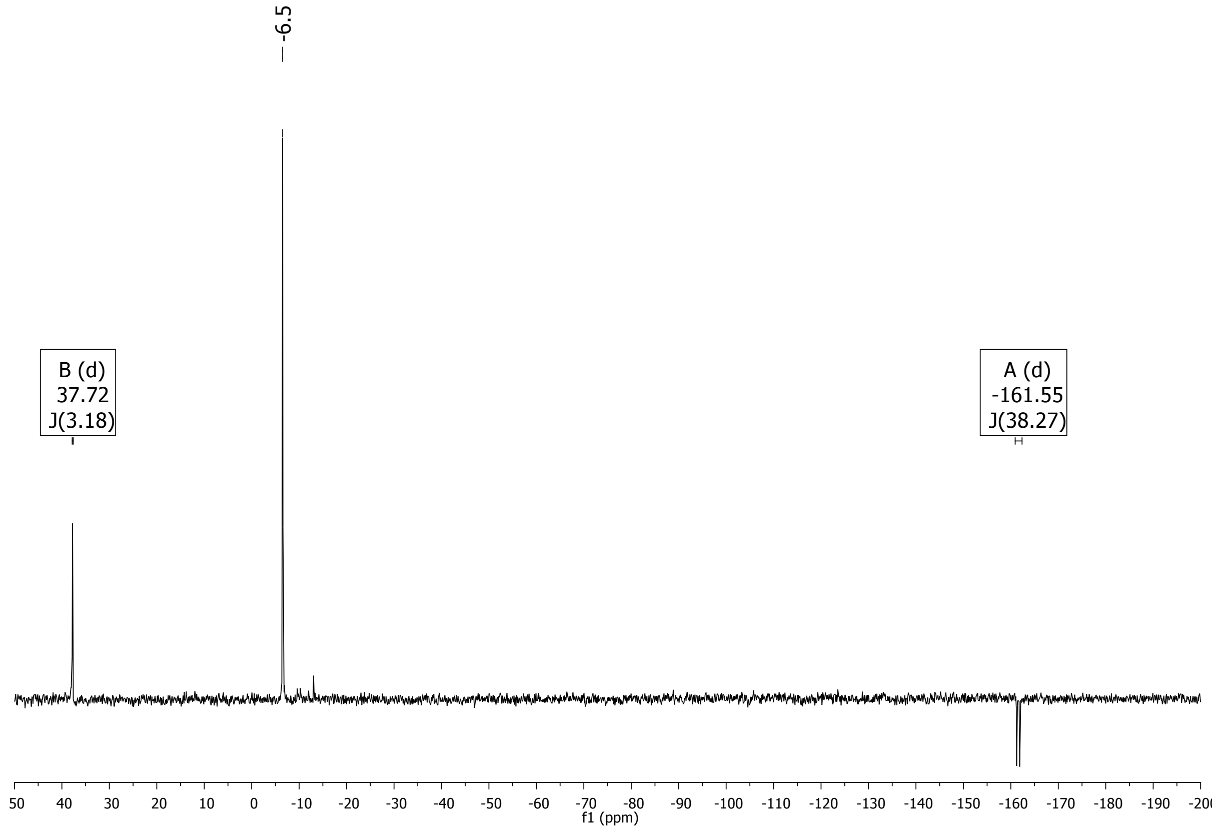

Figure S10. ^{29}Si*(DEPT) NMR spectrum of* ***2Y*** *in DME using a* D_2O *capillary for deuterium lock.*

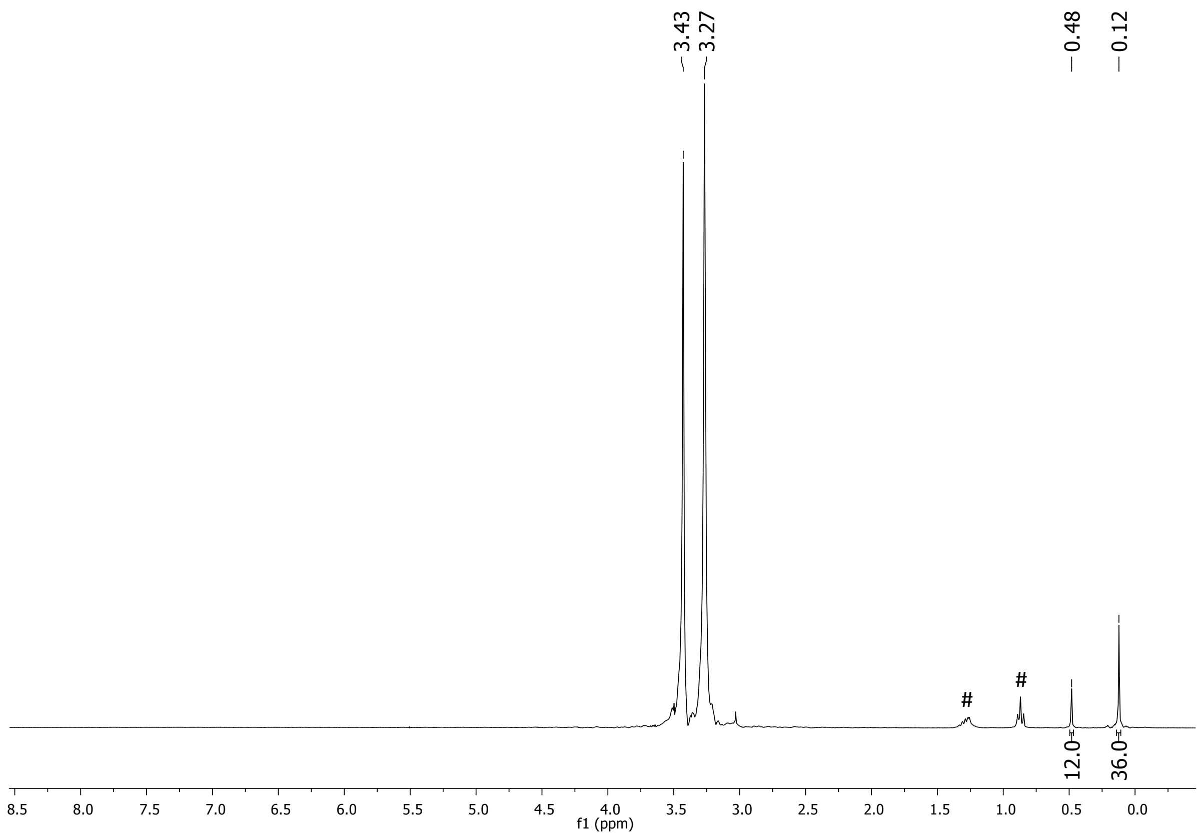

Figure S11. *^{1}H NMR spectrum of **2La** in DME using a D_2O capillary for deuterium lock. Solvent related signals marked as following: pentane (#).*

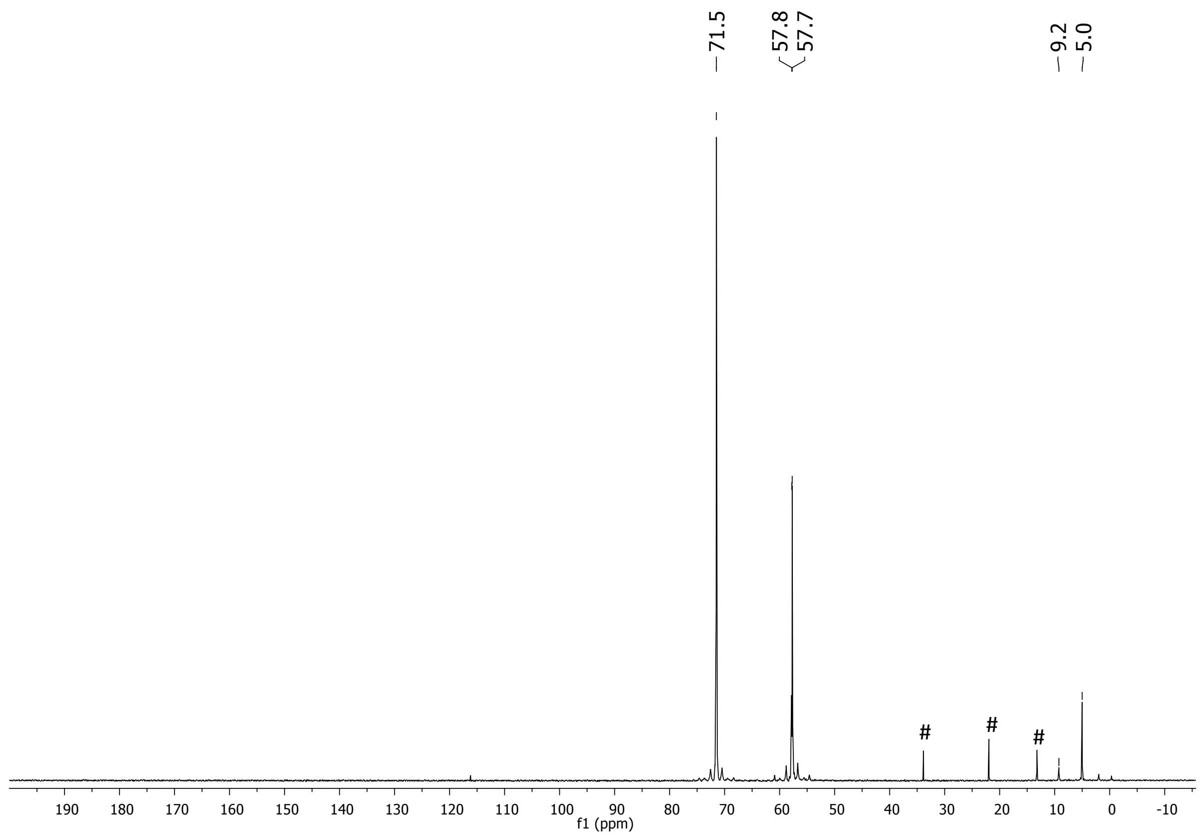

Figure S12. *^{13}C NMR spectrum of **2La** in DME using a D_2O capillary for deuterium lock. Solvent related signals marked as following: pentane (#).*

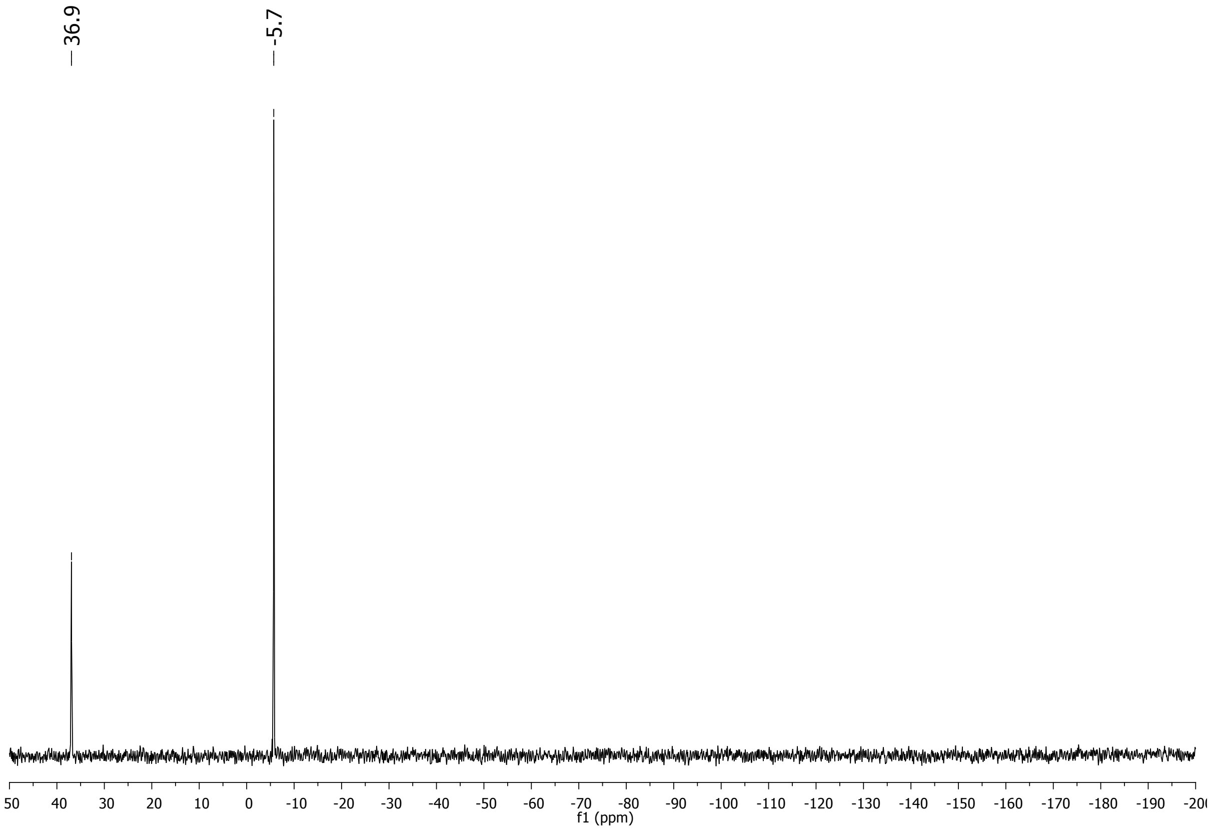

Figure S13. *^{29}Si(DEPT) NMR spectrum of **2La** in DME using a D_2O capillary for deuterium lock.*

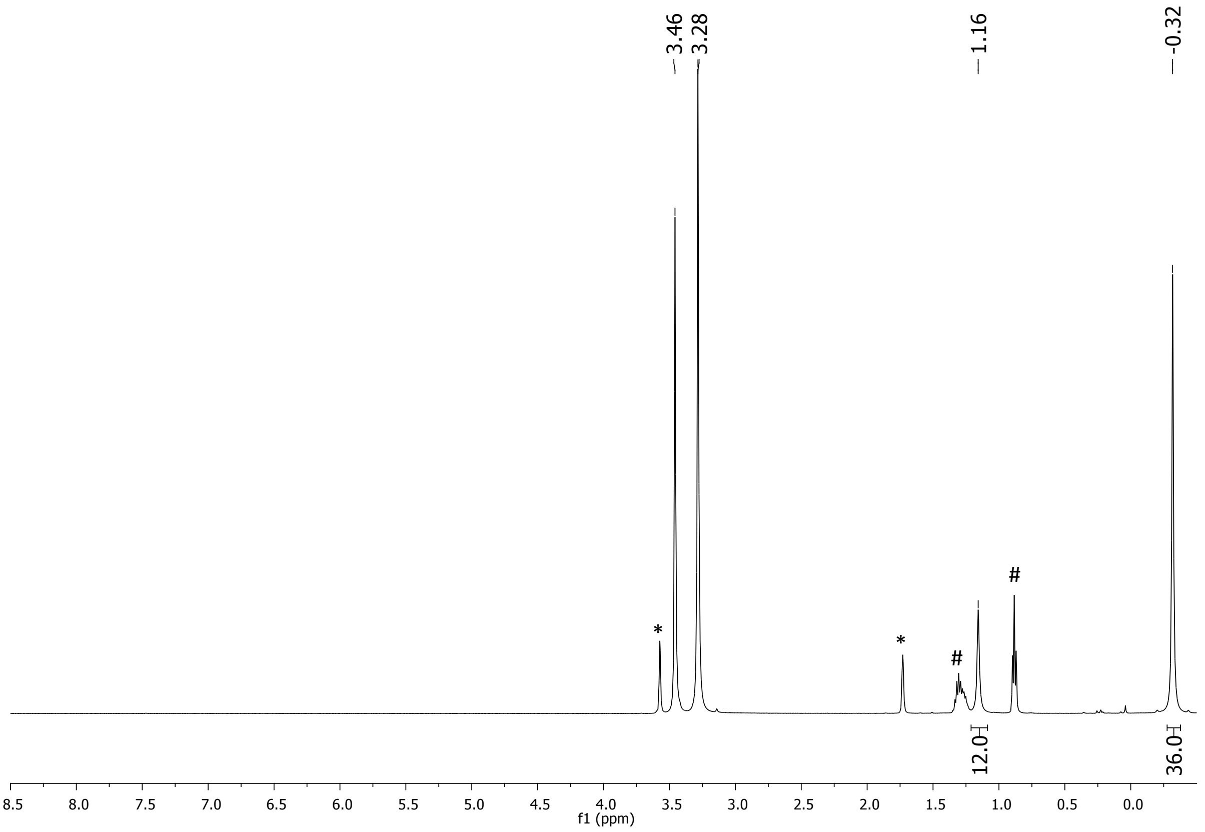

FigureS14. *^{1}H NMR spectrum of* ***2Ce*** *in d^{8}-THF. Solvent related signals marked as following: d^{8}-THF (*), pentane (#).*

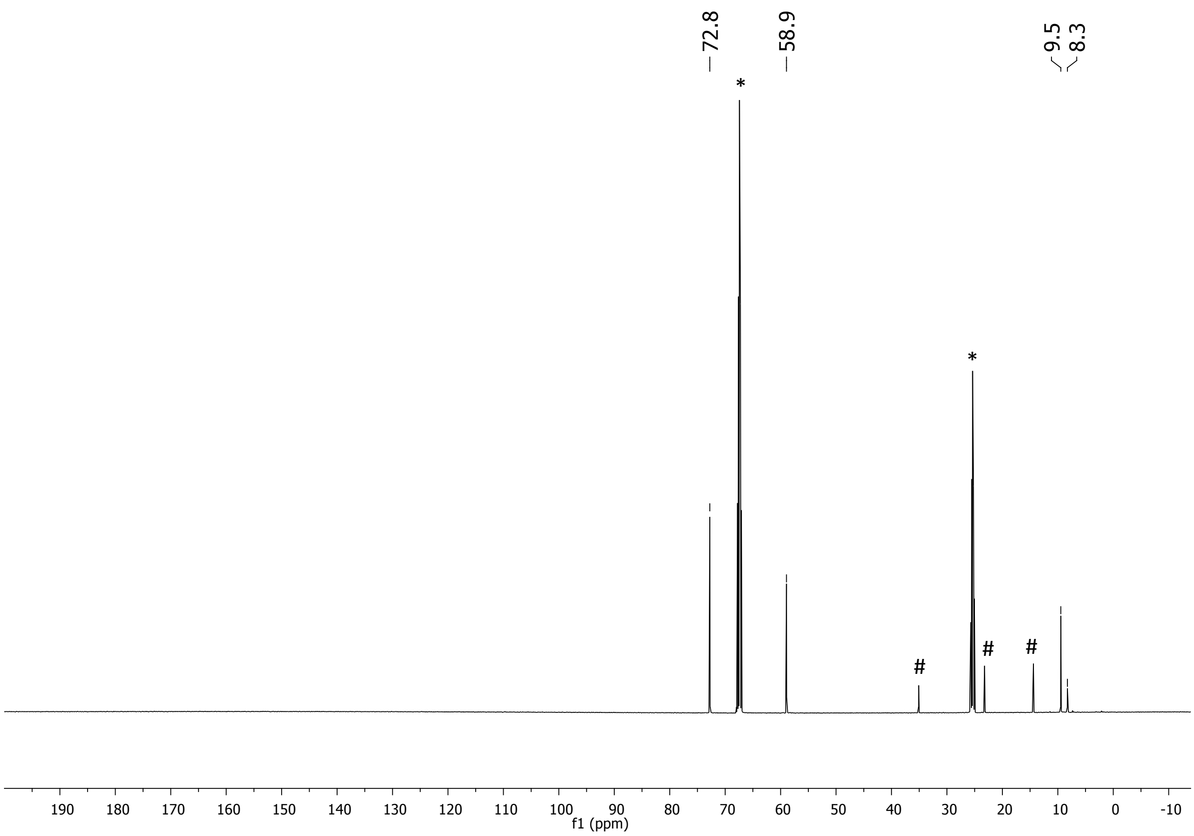

Figure S15. *^{13}C NMR spectrum of* ***2Ce*** *in d^8-THF. Solvent related signals marked as following: d^8-THF (*), pentane (#).*

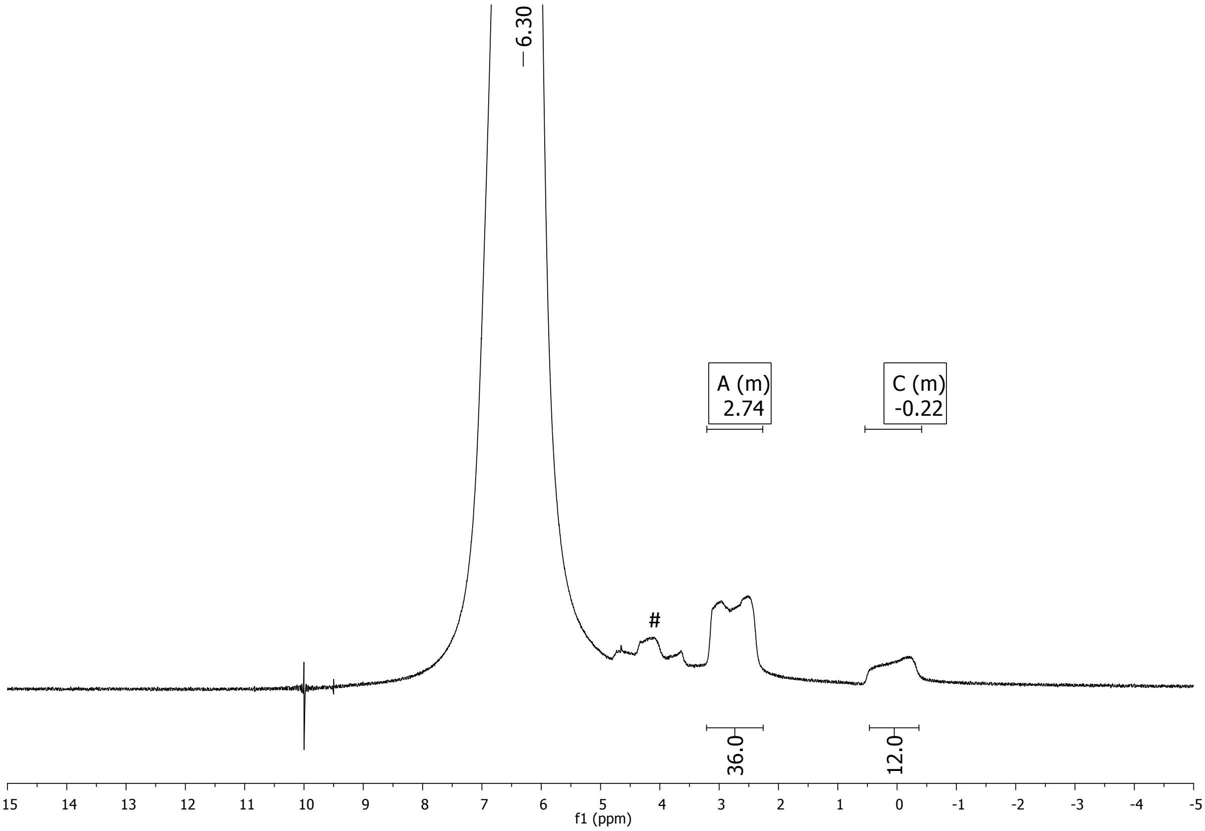

Figure S16. *^{1}H NMR spectrum of **2Pr** in DME using a D_2O capillary for deuterium lock. Solvent related signals marked as following: pentane (#).*

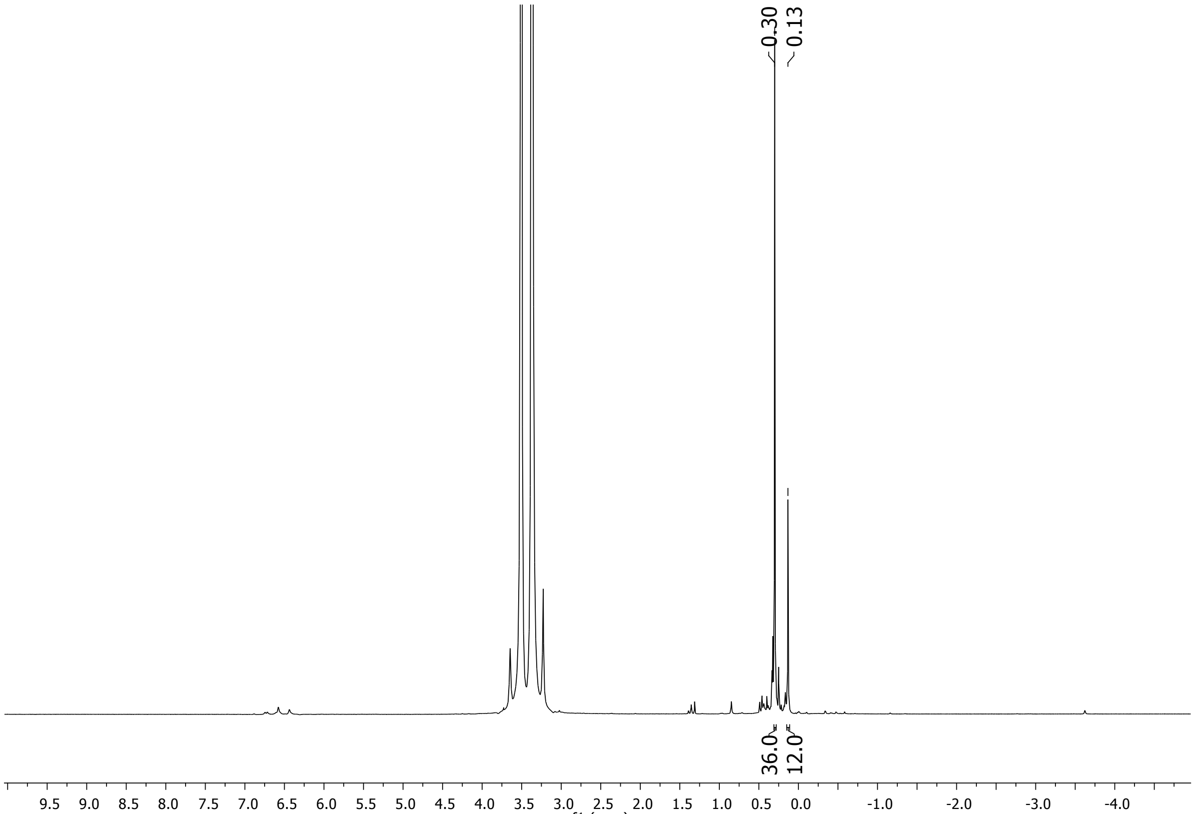

Figure S1. *^{1}H NMR spectrum of **2Sm** in DME using a D_2O capillary for deuterium lock.*

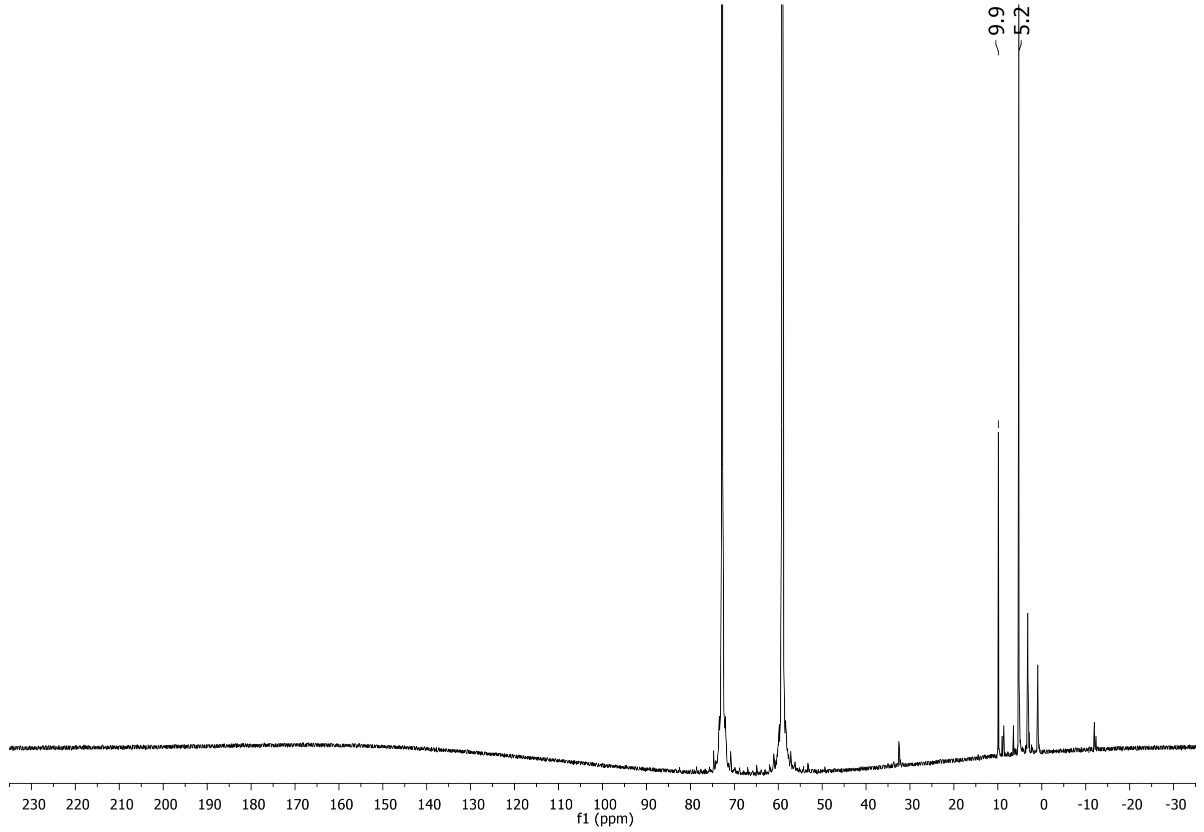

Figure S2. *^{13}C NMR spectrum of **2Sm** in DME using a D_2O capillary for deuterium lock.*

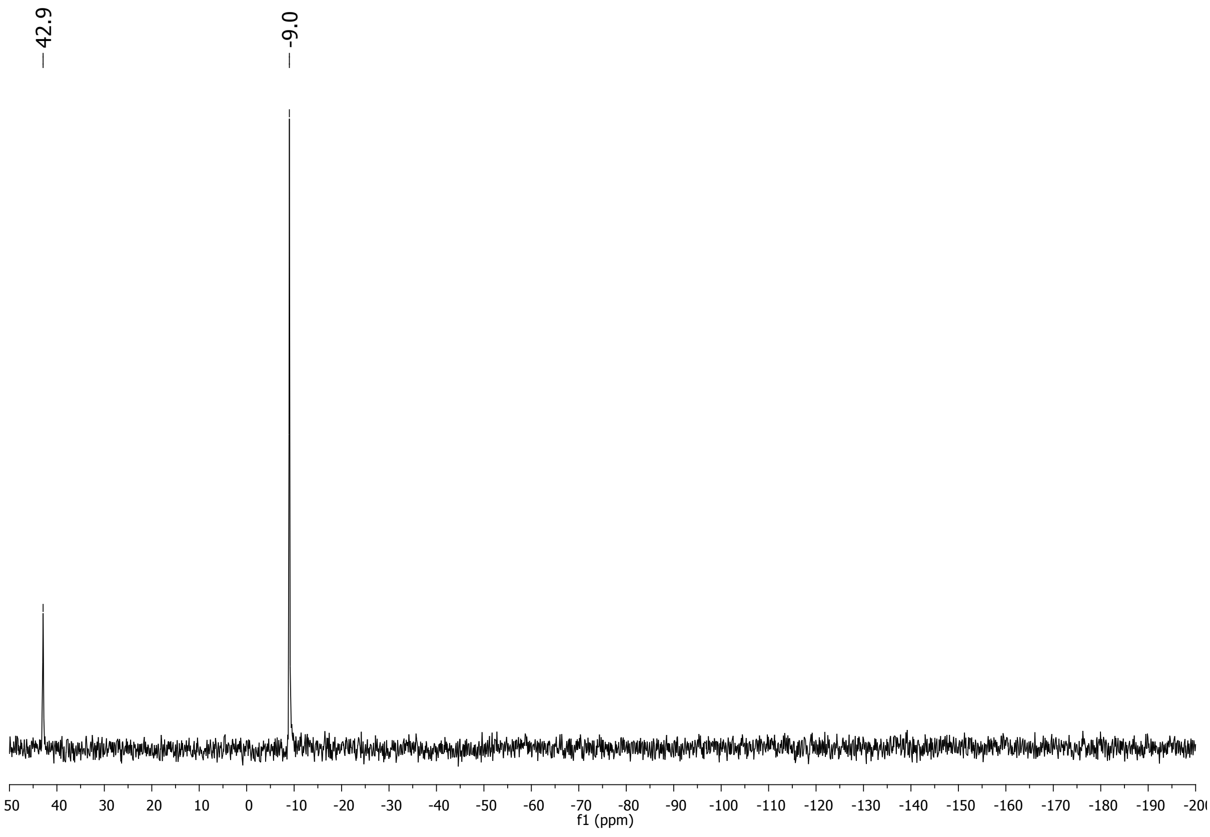

Figure S3. *^{29}Si(DEPT) NMR spectrum of **2Sm** in DME using a D_2O capillary for deuterium lock.*

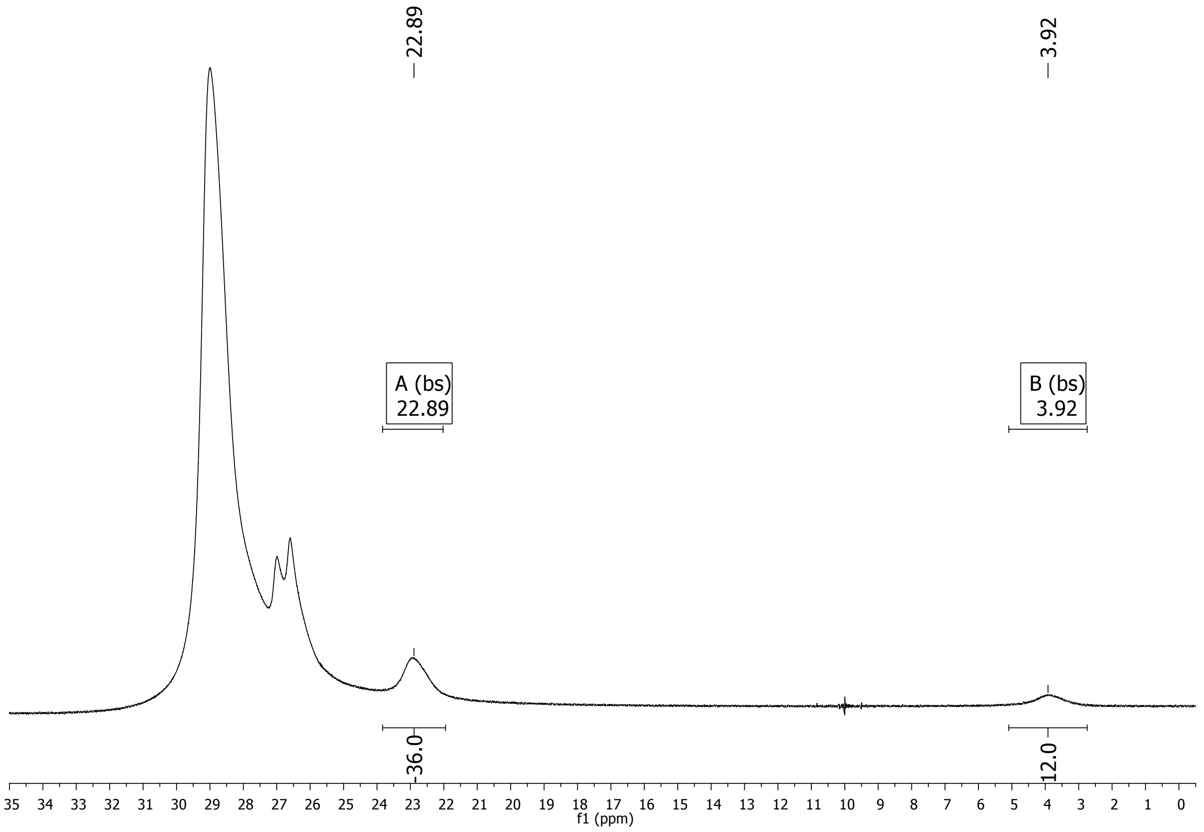

Figure S20. *^{1}H NMR spectrum of **2Tb** in DME using a D_2O capillary for deuterium lock.*

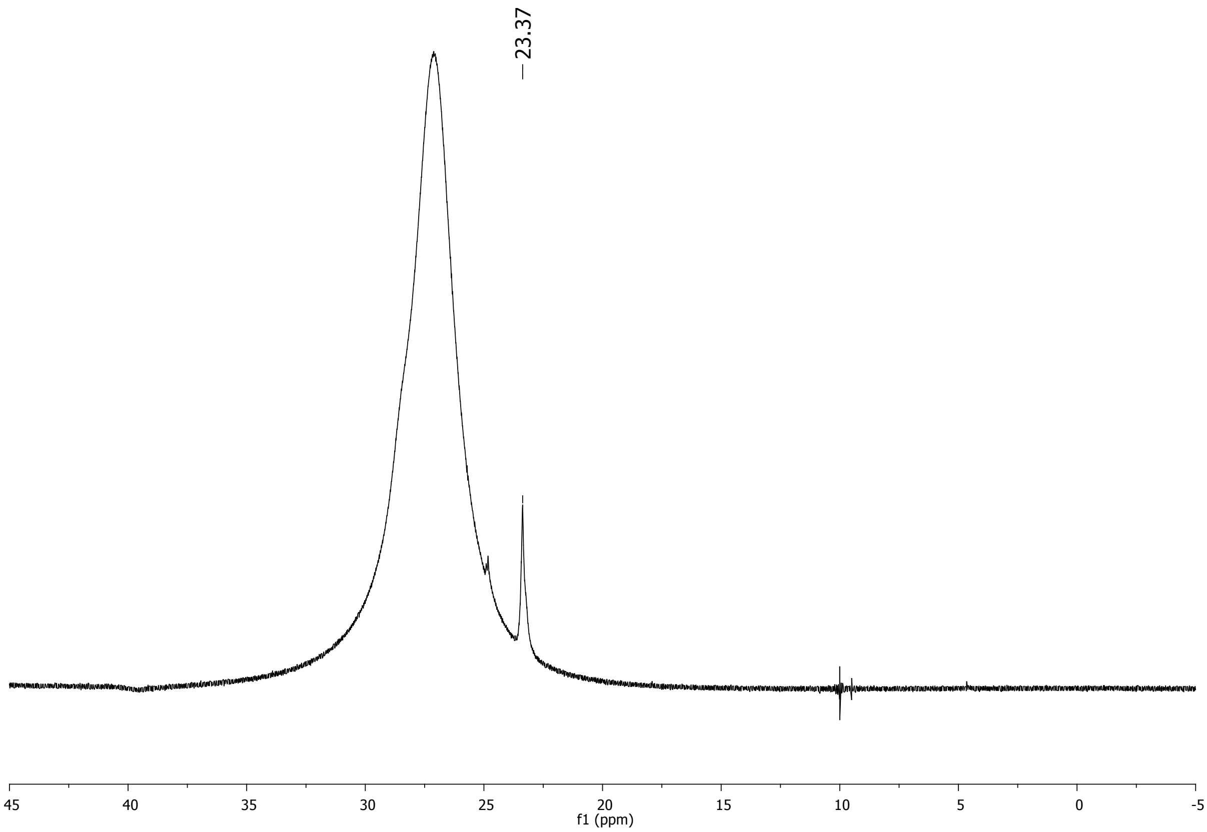

Figure S21. *^{1}H NMR spectrum of **2Dy** in DME using a D_2O capillary for deuterium lock.*

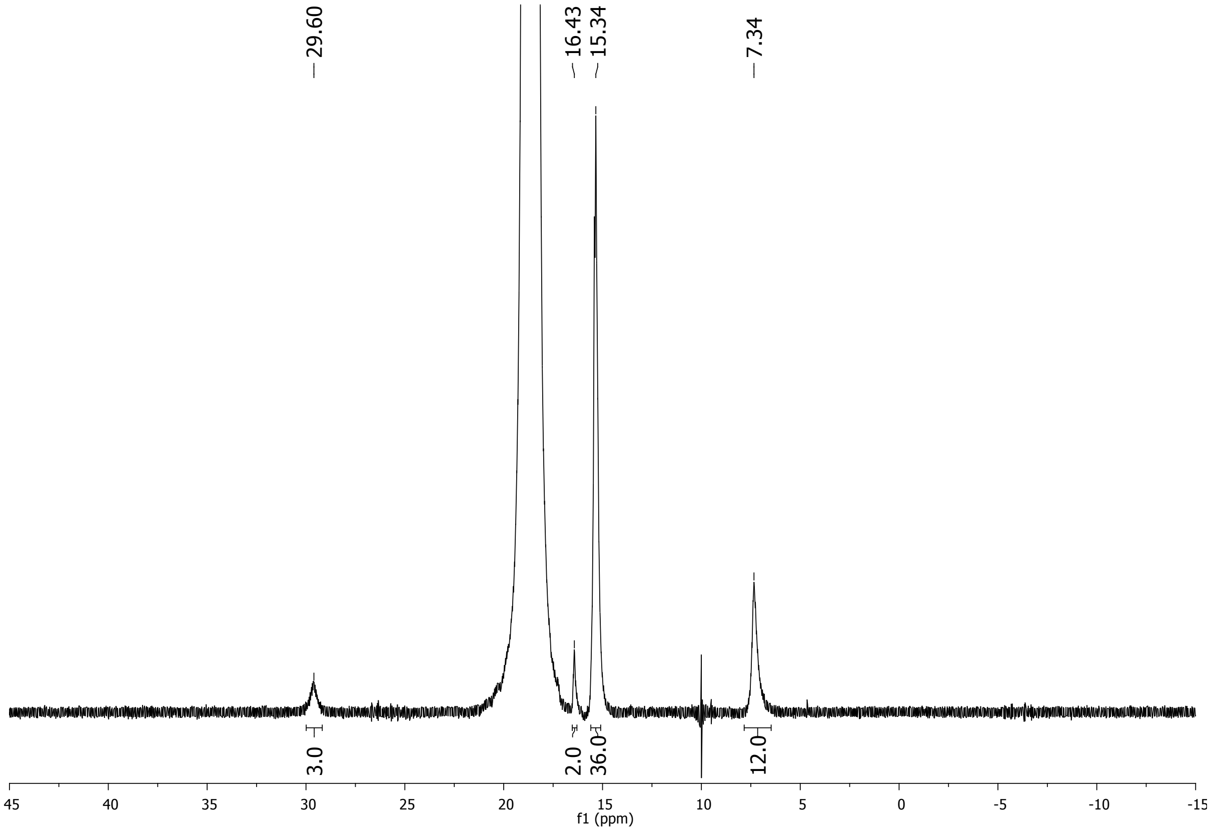

Figure S22. *^{1}H NMR spectrum of **2Er** in DME using a D_2O capillary for deuterium lock.*

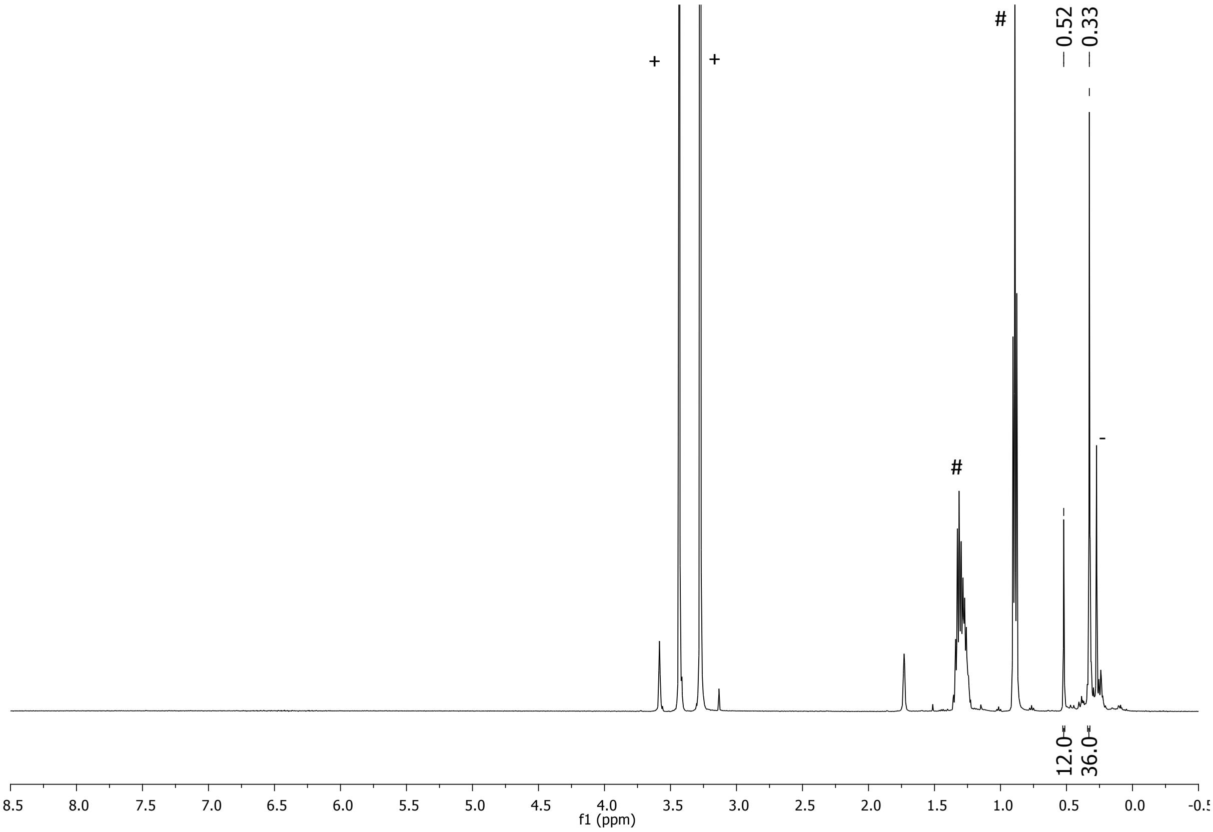

Figure S23. *^{1}H NMR spectrum of **4** in d^8-THF. Other related signals marked as following: pentane (#), DME (+), 2,2,5,5-tetramethyl-3,3,4,4-tetrakis(trimethylsilyl)-1-oxacyclopentasilane (-).*

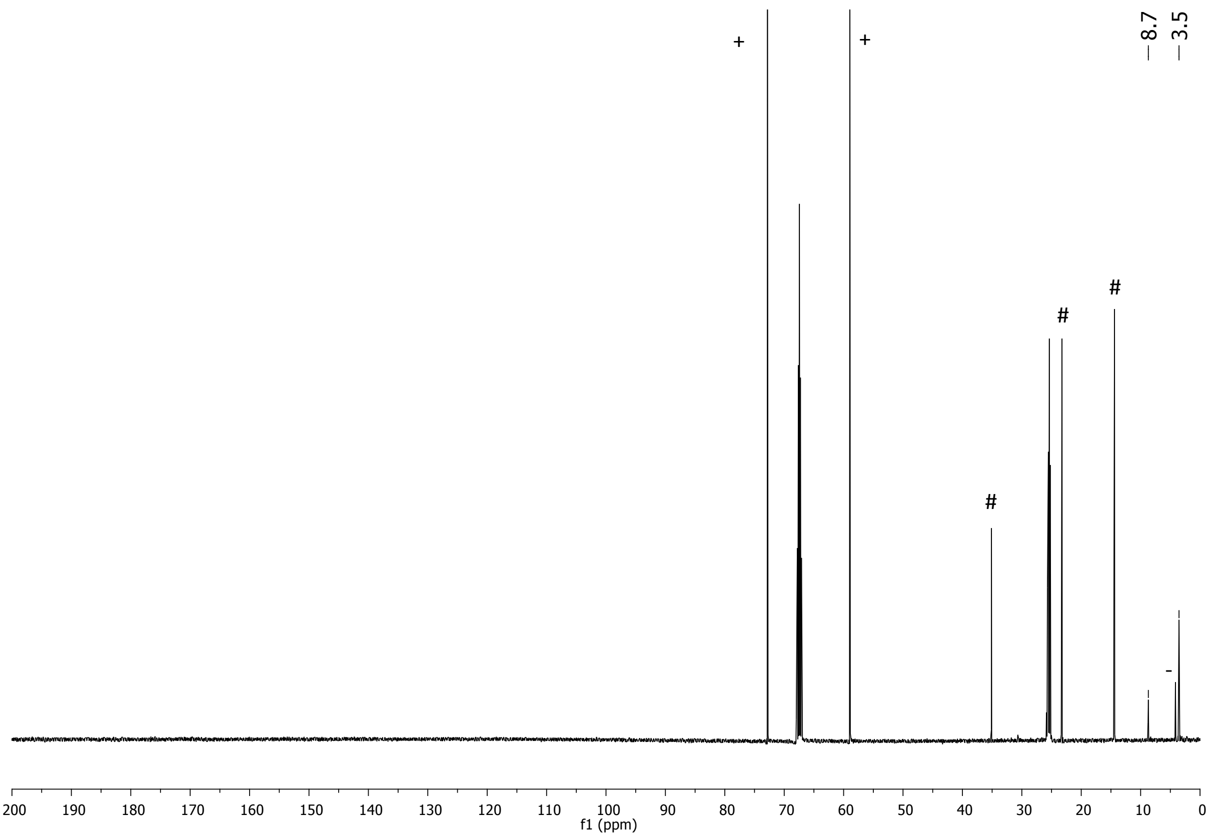

Figure S24. ^{13}C NMR spectrum of **4** in d^8-THF. Other related signals marked as following: pentane (#), DME (+), 2,2,5,5-tetramethyl-3,3,4,4-tetrakis(trimethylsilyl)-1-oxacyclopentasilane (-).

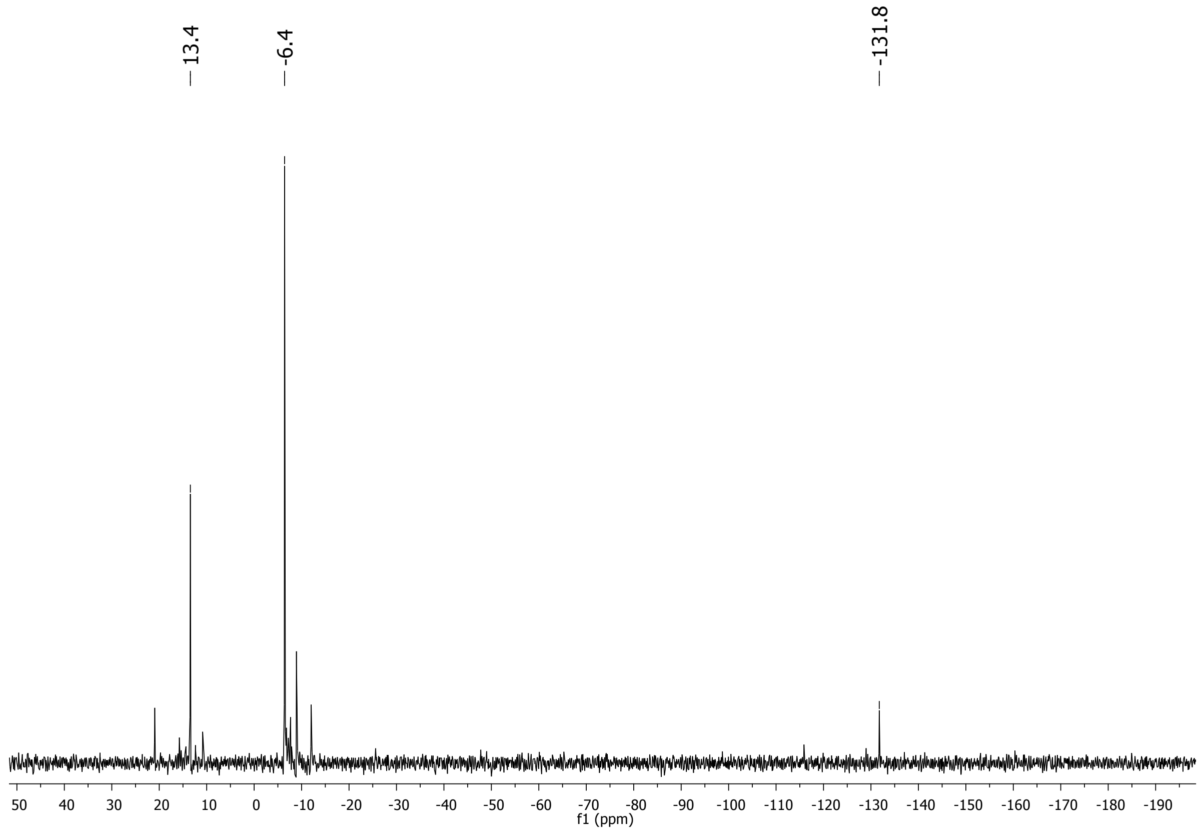

Figure S25. ^{29}Si(DEPT) NMR spectrum of **4** in DME using a D_2O capillary for deuterium lock.

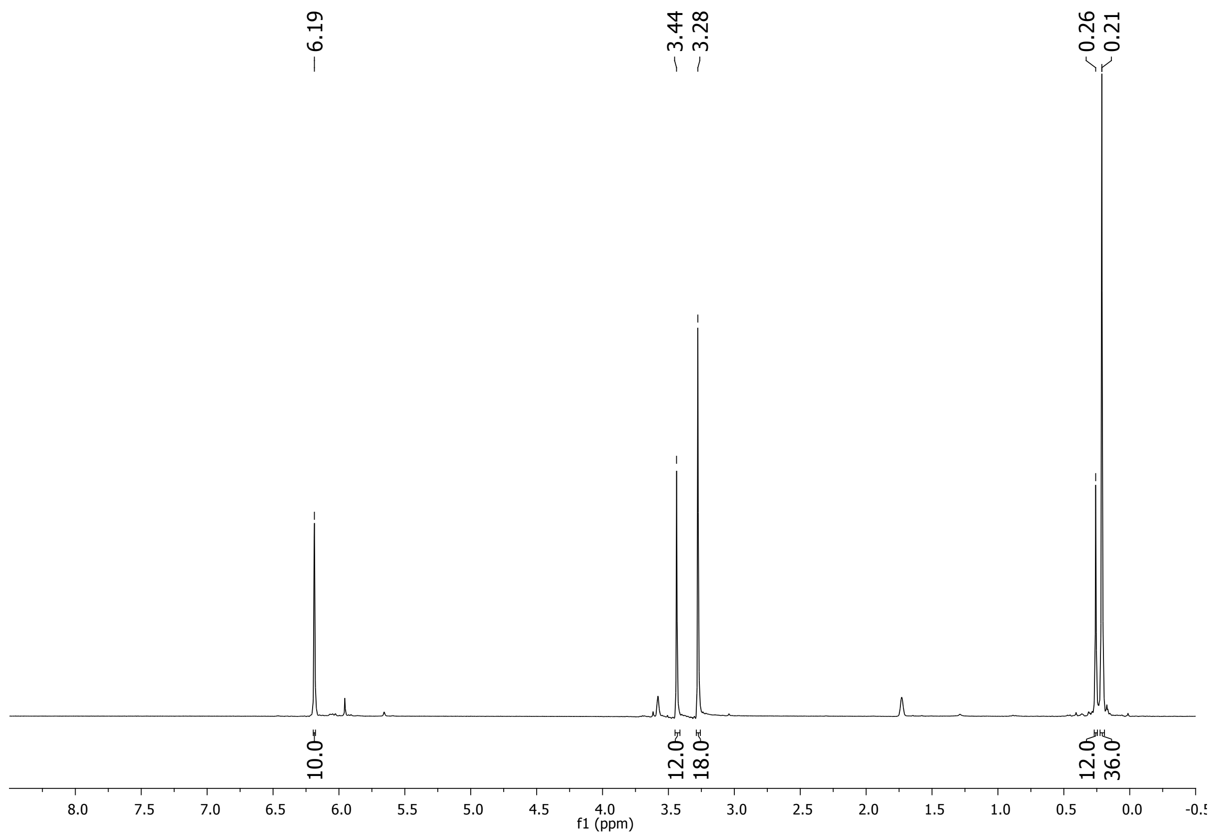

Figure S26. *¹H NMR spectrum of* ***5*** *in d⁸-THF.*

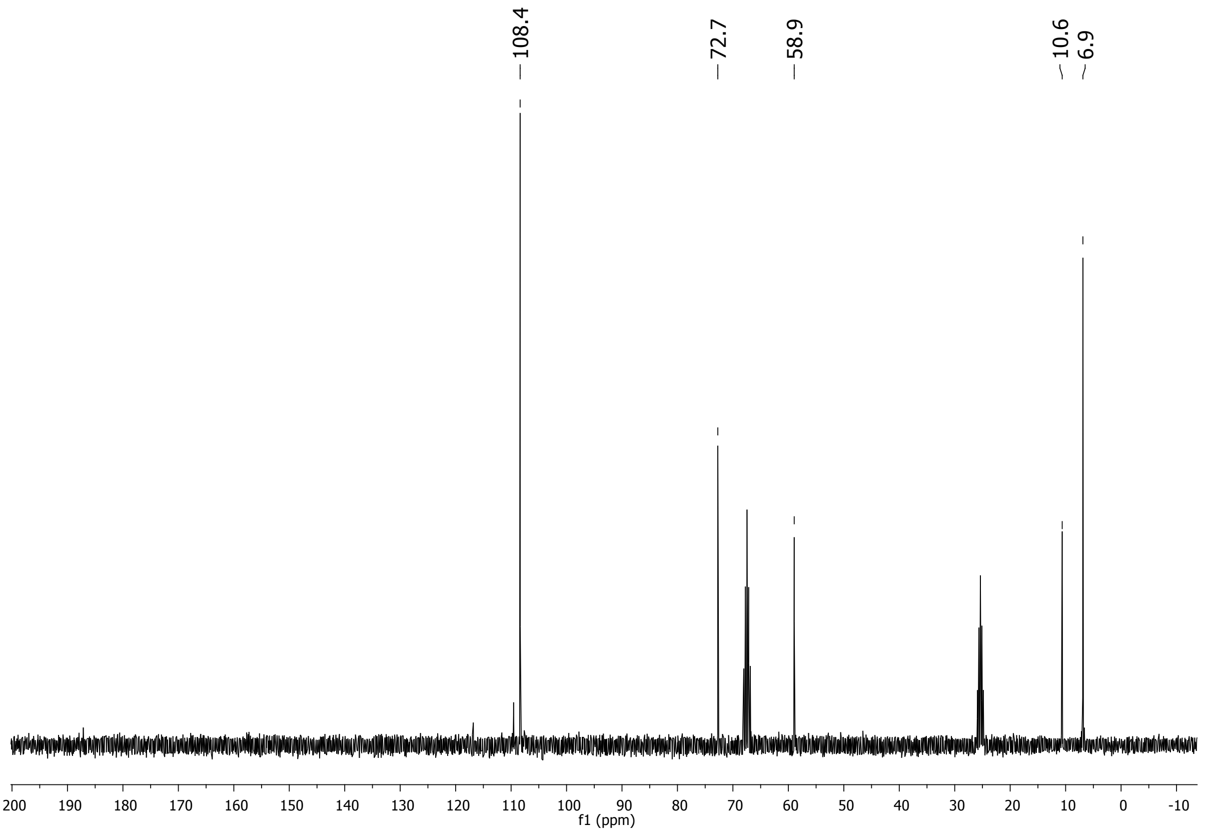

Figure S27. *¹³C NMR spectrum of* ***5*** *in d⁸-THF.*

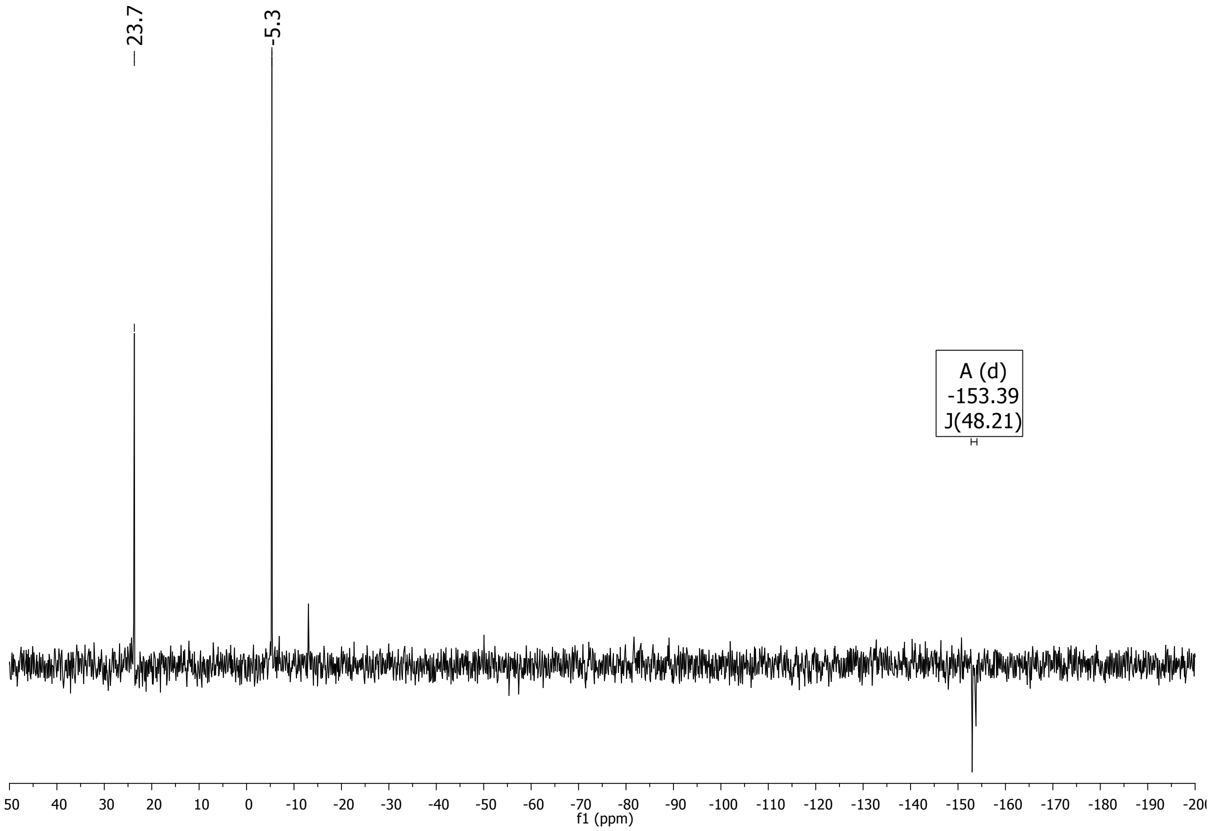

Figure S28. *^{29}Si(DEPT) NMR spectrum of **5** in DME using a D_2O capillary for deuterium lock.*
